# Data Integration in Poplar: ‘Omics Layers and Integration Strategies

**DOI:** 10.3389/fgene.2019.00874

**Published:** 2019-09-25

**Authors:** Deborah Weighill, Timothy J. Tschaplinski, Gerald A. Tuskan, Daniel Jacobson

**Affiliations:** ^1^The Bredesen Center for Interdisciplinary Research and Graduate Education, University of Tennessee, Knoxville, Knoxville, TN, United States; ^2^Biosciences Division, Oak Ridge National Laboratory, Oak Ridge, TN, United States

**Keywords:** data integration, *Populus*, multi-omic data, networks, signal processing, wavelet transform

## Abstract

*Populus trichocarpa* is an important biofuel feedstock that has been the target of extensive research and is emerging as a model organism for plants, especially woody perennials. This research has generated several large ‘omics datasets. However, only few studies in *Populus* have attempted to integrate various data types. This review will summarize various ‘omics data layers, focusing on their application in *Populus* species. Subsequently, network and signal processing techniques for the integration and analysis of these data types will be discussed, with particular reference to examples in *Populus*.

## Introduction

Poplar species (*Populus* sp.) are promising sources of cellulosic biomass for biofuels because of their fast growth rate, high cellulose content, and moderate lignin content (Sannigrahi et al., 2010). Ragauskas et al. (2006) outline areas of research needed “to increase the impact, efficiency, and sustainability of biorefinery facilities,” such as research into modifying plants to enhance favorable traits, including altered cell wall structure leading to increased sugar release, as well as resilience to biotic and abiotic stresses. One particular research target in *Populus* is the decrease/alteration of the lignin content of cell walls. The *Populus* genus contains a considerable amount of variation, is estimated to contain approximately 30 different species (Taylor, 2002), and is considered a model species for trees and woody species. Major *Populus* species and their characteristics can be found in the review by Taylor (2002).

There is an increasing movement towards integrating multiple layers of ‘omics data in a systems biology approach to understand gene–phenotype relationships and assist in plant breeding programs [see Ingvarsson et al. (2016), Weckwerth (2011), and Valledor et al. (2018) for reviews]. Increasing systems biology knowledge through data integration in *Populus* species represents an important step in the development of *Populus* as a model system, as well as an efficient feedstock for biofuels through selective breeding programs and accelerated domestication (Tuskan, 2007). This review will discuss different sources of ‘omics data layers with a particular focus on those previously applied in *P. trichocarpa* or other *Populus* species. We will also briefly mention ‘omics layers that have not yet been applied in *Populus* species, but would be useful tools to consider to extend the systems biology knowledge of *Populus* species. Subsequently, we will review network and signal processing approaches to representing, analyzing, and integrating multiple ‘omics data layers, again providing examples in *Populus* species where possible.

## Sources of ‘Omics Data Layers

### Overview

Different ‘omics layers each provide information on a different aspect of a complex biological system (**Table 1**). Below, we discuss different ‘omics layers and the information they can provide about the system. We also present examples each of the ‘omics data layers in the literature, focusing on examples in *Populus* species where available.

**Table 1:** ‘Omics data layers.

‘Omics Data layer	Information gained
Genomics	Primary DNA sequence, gene annotations, transposable elements, repetitive sequences, genome variants
Transcriptomics	Gene expression, mRNA abundances, gene co-expression, potential gene co-regulation, response of organism (cell, tissue) to different conditions at the mRNA level
Metabolomics	Metabolite abundances, response of organism to different conditions at the metabolite level
Proteomics	Protein abundances, post-translational modifications, response of organism to different conditions at the protein level
GWAS	Associations between genomic variants and phenotypes in a population, potential pleiotropic/epistatic relationships
Epigenomics	Epigenetic features such as DNA methylation, chromatin accessibility
DAP-Seq	Transcription factor-DNA binding

### Genomics

#### Genome and Annotation

The genome sequence of Black Cottonwood, *Populus trichocarpa* (Torr. & Gray) was released in 2006 (Tuskan et al., 2006). This genome, a single female genome “Nisqually-1,” was the first tree to have its complete genome sequenced, and it became a model system for studies on woody perennial plants (Wullschleger et al., 2012; Jansson and Douglas, 2007). The *P. trichocarpa* genome consists of 19 chromosomes, with chromosome 19 predicted to be evolving into a sex chromosome (Yin et al., 2008). Analysis of homologous regions of the genome showed evidence for several whole-genome duplication events; the most recent being the Salicoid duplication event, which is contained within the family *Salicaceae*, the next termed the *Eurosid* duplication shared among *Eurosids*, and an ancient duplication event shared by all land plants (Tuskan et al., 2006).

Since initial sequencing, the genome assembly has gone through several revisions and is now in its fourth version. Furthermore, a genome-wide association study (GWAS) population of ∼1,000 natural accessions from the United States and Canada was propagated in multiple common gardens and resequenced, providing a rich resource for studies of the variation in natural *P. trichocarpa* populations as well as GWASs (Tuskan et al., 2011; Slavov et al., 2012; Evans et al., 2014).

The genome sequence is available on Phytozome (Goodstein et al., 2012), and the genome along with gene and functional annotation such as Gene Ontology (GO) terms and PFams can be viewed and interacted with using the JBrowse (Skinner et al., 2009) plugin on Phytozome (https://phytozome.jgi.doe.gov/pz/portal.html).

This genomic data layer provides a base on which many other data sources can be layered, including various annotations and features of the genomic sequences as well as other data layers downstream in the central dogma of molecular biology.

#### Genomic Variants

Different individuals in a population can accumulate variation in their genome, such as single-nucleotide polymorphisms (SNPs) involving a nucleotide change at a single position, insertions/deletions of a single nucleotide or larger pieces of DNA, copy number variations (CNVs) of DNA segments, or translocations (the movement of a section DNA from one location to another) (Abel and Duncavage, 2013).

There are two major approaches to calling SNPs in a given sample in a relatively high-throughput manner, namely, a genotyping SNP array and SNP calling from next-generation sequencing (NGS) data. A genotyping SNP array involves hybridizing extracted DNA to an array containing probes with known SNPs (LaFramboise, 2009) and is thus limited by the SNPs chosen to appear on the array. For example, the *P. trichocarpa* genotyping array is based on 34,131 SNPs located near/within around 3,500 selected candidate genes (Geraldes et al., 2013). SNP calling through NGS involves whole-genome shotgun sequencing of all individuals, aligning of all reads to a common reference genome, and then calling variants (Nielsen et al., 2011) using software such as GATK (McKenna et al., 2010). The advantage of SNP calling from NGS data is that one is not limited by the set of SNPs available on an array. A larger number of SNPs can be detected, and the discovery of new SNPs is possible. SNP genotyping arrays are also not able to detect other classes of genome variants such as translocations and inversions (LaFramboise, 2009).

A population of ∼1,000 natural *P. trichocarpa* accessions have been clonally propagated in four common gardens (Tuskan et al., 2011) and resequenced in order to provide NGS data for SNP calling. Several studies have been published, making use of SNPs called across parts of this population (Slavov et al., 2012; Evans et al., 2014; McKown et al., 2014). Slavov et al. (2012) performed a study involving SNPs called from resequenced genomes of 16 of the genotypes within this *P. trichocarpa* population. PCA analysis of SNP genotypes revealed clear separation based on the geographic origin of the genotypes and linkage disequilibrium was reported to decay to *r^2^* ≤ 0.2 within 3–6 kb. It is important to note that this is based on the resequencing of only 16 genotypes. A set of ∼28 million bi-allelic SNPs called across 882 genotypes from this population have been publicly released and are available online from DOI https://doi.org/10.13139/OLCF/1411410.

#### Genotype–Phenotype Associations

Phenotypes are often complex traits, in that they are influenced or controlled by a great number of genes (Solovieff et al., 2013). GWASs attempt to associate the presence/absence of SNPs with these complex traits (Visscher et al., 2012;Solovieff et al., 2013). This involves genotyping a large sample of individuals of a population, measuring phenotypes across these individuals and statistically determining the association between the presence/absence of the genotyped markers or SNPs and the phenotypes across the population (Korte and Farlow, 2013). A general concern when conducting GWASs is that individuals within a population that are genetically related can share both causal alleles, which impact the phenotype (Visscher et al., 2012), and non-causal alleles (Korte and Farlow, 2013). These causal and non-causal alleles could be located nearby to each other on the chromosome and could thus be in linkage disequilibrium (LD)—alleles that are correlated across a population and thus co-inherited (Flint-Garcia et al., 2003). This LD between causal and non-causal alleles across related individuals could result in non-causal alleles being correlated with a phenotype when they have no actual effect on the phenotype.

GWAS analyses generally require that the individuals in the population are unrelated. However, some level of population structure due to shared ancestors can cause spurious associations between genotype and phenotype, and accounting for population structure is thus important in order to remove variance that is due solely to the relatedness of individuals (for a useful review, see Astle and Balding et al., 2009). It is thus important to account for population structure in association models. However, there is the possibility of masking true associations that happen to correlate with population structure because they are local adaptations of clades to local environments.

An important element of GWAS studies is the issue of multiple hypothesis correction, as a GWAS typically involves the calculation of thousands or millions of statistical tests. An exhaustive review of different GWAS approaches and multiple hypothesis correction is beyond the scope of this review; however, we discuss these topics briefly in the **Supplementary Text 1**, and we refer the readers to useful articles on these topics (Noble 2009; Johnson et al., 2010; Bush and Moore, 2012; Korte and Farlow, 2013; Fadista et al., 2016; Visscher et al., 2017).

Several studies involving GWAS analyses in *P. trichocarpa* have been published. McKown et al. (2014) genotyped 448 individuals from the *P. trichocarpa* GWAS population using an SNP array containing ∼34,000 SNPs and performed GWAS on 40 different traits measured in the population. These traits included biomass phenotypes such as height, volume, and height:diameter ratio; ecophysiological traits such as leaf shape, chlorophyll content, and carbon:nitrogen ratio; and phenology traits such as bud set, growth period, and leaf drop. A set of 1118 significant GWAS associations were identified involving 410 unique SNPs, 78% of which occurred in non-coding regions and 28% occurred in coding regions. This resulted in 275 genes having significant trait associations, many of which were transcription factors or regulators of some kind. A subset of 42 of the 275 genes exhibited multiple GWAS associations with traits in different trait categories, exhibiting potential pleiotropy.

Evans et al. (2014) used whole-genome sequencing of 544 individuals from the *P. trichocarpa* GWAS population, and subsequent variant calling identified 17,902,740 SNPs. They found that 1) nucleotide diversity was twice as high in intergenic space than in genic space, 2) diversity was even lower in coding space, and 3) a large proportion of the SNPs had a minor allele frequency (MAF) ≤ 0.01 and were thus considered rare alleles. Metrics of natural selection, such as *F_ST_*, were used to identify candidate regions under strong selection and suggest that this could be driven by climate.

Tuskan et al. (2018) tested callus induction in 280 genotypes from within the *P. trichocarpa* GWAS population and performed a GWAS analysis to identify SNPs potentially affecting callus formation. Eight genes potentially associated with callus formation were identified. Combining GWAS results with co-expression information allowed for a putative regulatory network for callus formation to be constructed.

In a recent study by Liu et al. (2018), 64 individuals from a full-sib family from a cross between *P. deltoides* and *P. euramericana* were genotyped using real-time PCR. Phenotypes used were stem heights and diameters over 24 years. Both a standard GWAS and distance correlation sure independence screening (DC-SIS) association tests are performed. DC-SIS is an association method that allows for a multi-dimensional phenotype (diameter measurements over time) as opposed to a single phenotype measurement.

#### Repetitive and Transposable Elements

Transposable elements (TEs) are segments of DNA that are mobile, in the sense that they can move from one genomic location to another. Type I elements, or retrotransposons, require an RNA intermediate, which is then reverse-transcribed into the genome at a different location (Slotkin and Martienssen, 2007; Wicker et al., 2007). This is thus a “copy and paste” mechanism. Type II TEs are called DNA transposons and involve the excision of the DNA TE and subsequent integration elsewhere. This can thus be described as a “cut and paste” mechanism (Slotkin and Martienssen, 2007; Wicker et al., 2007). Many TEs are no longer active because mutations have inhibited their ability to transpose. However, some TEs are silenced by the host. This can include mechanisms such as silencing by RNAi or though DNA/histone methylation (Slotkin and Martienssen, 2007).

Different TEs show preference for insertion at different locations in the genome, and thus exhibit very different distributions across the genome (Bennetzen and Wang, 2014). TEs have large impacts on genome characteristics and evolution (Klein and O’Neill, 2018). Firstly, they have a significant impact on genome size, comprising a large part of many plant genomes (Bennetzen and Wang, 2014), ranging from 10% of the genome of *Medicago truncatula*, 42% of *P. trichocarpa*, and 80% of *Pinus taeda* (loblolly pine) (Kejnovsky et al., 2012). Unequal homologous recombination can also result from the presence of multiple TEs of a given family. This can cause various genome rearrangements including duplications, inversions, deletions, and translocations (Gaut et al., 2007;Bennetzen and Wang, 2014). TEs that insert into gene regions can cause the gene to become non-functional. In addition, TEs that insert near genes can impact the expression pattern of the genes, especially since some TEs contain regulatory sequences (Wicker et al., 2007; Bennetzen and Wang, 2014). Application of stress to an organism has been shown to activate TEs, leading to the hypothesis that TEs create variability in the genome that could be useful under times of stress (Capy et al., 2000).

Since the genome release, several investigations of repeats and TEs have been performed in *P. trichocarpa*. Soon after the release of the *P. trichocarpa* genome, Zhou and Xu (2009) annotated repeat sequences in the genome and made them publicly available in a database called RepPop. However, this database no longer appears to be available. Cossu et al. (2012) identified LTR repeats in *P. trichocarpa* and investigated their distribution across the genome, finding Gypsy LTRs to be enriched in putative centromeric regions. Soon after, Natali et al. (2015) surveyed LTR retrotransposons in an updated version of the *P. trichocarpa* genome. Vining et al. (2012) investigated the number of repeats and genes that were methylated vs non-methylated in *P. trichocarpa*, and found that methylated retroelements, LTRs, hAT elements, CACTA elements, and certain LINEs were overrepresented when compared to their un-methylated versions. It was also found that the methylation patterns of TEs differed significantly across tissues (Vining et al., 2012). Usai et al. (2017) performed an investigation into the repetitive DNA content of seven different *Populus* species, including *P. deltoides*, *P. nigra*, *P. tremula*, *P. tremuloides*, *P. balsamifera*, *P. simonii*, and *P. trichocarpa*. LTR repeats were the dominant repeat type across all species, although the total repeat content varied from 33.8% in *P. nigra* to 46.5% in *P. tremuloides*.

In a recent study by Mascagni et al. (2018), insertion ages of LTR TEs were determined in *P. trichocarpa* by comparing the sequences of the 3′ and 5′ ends of LTRs. This provides an indication of the time since insertion because at the time of insertion, the 3′ and 5′ LTRs are identical, and subsequently accumulate mutations independently after insertion. Insertion time was also determined by comparing the sequences of paralogous RTs from the same lineages. The two methods provided conflicting results, with the LTR comparison method suggesting that Gypsy TEs were older than Copia TEs, whereas the RT comparison method did not find a significant difference in the age of these classes. Yi et al. (2018) recently published a database (SPTEdb) of TEs in *P. trichocarpa*, *P. euphratica*, and *Salix suchowensis*. This database provides TE annotation for these organisms using multiple TE identification methods and presents these in a database format as well as a JBrowse interface.

### Transcriptomics

Transcriptomic analysis involves the measuring of the expression levels of messenger RNA. Various study designs have been implemented in *P. trichocarpa* to investigate a variety of properties of the cellular system. Several studies have focused on the response of the *Populus* transcriptome, or a subset of the transcriptome, to drought stress. The study by Shuai et al. (2013) used RNA-Seq to identify microRNAs responsive to drought stress, and subsequently, Shuai et al. (2014) performed RNA-Seq on control and drought leaf samples of *P. trichocarpa* to identify long intergenic non-coding RNAs (lincRNAs) that were responsive to drought stress. Tang et al. (2015) used RNA-Seq to identify genes differentially expressed between well-watered and water-limited samples, and several differentially expressed genes and functions were identified. Genes related to energy metabolism and growth (cell division and tissue expansion) were significantly downregulated, and Potri.013G093600, a homolog of an *Arabidopsis thaliana* vacuolar pyrophosphatase (*AVP1*) was significantly upregulated. This gene had been previously found to improve drought and salt tolerance in several plants. Another transcriptomic drought study used Affymetrix microarrays for expression measurements of *P. tremula* × *P. alba* roots for six time points under drought stress. Differential expression and network analysis identified two interesting genes (PtaJAZ3 and PtaRAP2.6), which, when overexpressed under drought conditions, increased root growth (Dash et al., 2018).

Other transcriptomic studies in *Populus* have focused on variation in gene expression across tissues or across a population. In the study by Quesada et al. (2008), gene expression levels in *P. trichocarpa* were measured across five different tissues (roots, young leaves, mature leaves, nodes, and internodes) using NimbleGen microarrays. Genes with tissue-specific gene expression were identified, with stem samples having the highest number of tissue-specific genes. GO enrichment was used to determine the enriched functions of organ-specific genes. The expression of *P. trichocarpa* genes across organs was also compared to the expression of their *A. thaliana* orthologs across equivalent tissues, and the authors concluded that, while there were some similarities between expression patterns across these two species, significant diversification in gene expression regulation has occurred between orthologs. Shi et al. (2009) used quantitative real-time PCR (qPCR) to determine the expression level of 95 genes in the phenylpropanoid pathway in xylem, leaf, shoot, and phloem tissues, in order to determine the abundance and tissue specificity of genes potentially involved in monolignol biosynthesis. Bao et al. (2013) performed RNA-Seq of xylem tissue from 20 *P. trichocarpa* individuals from different populations, identified a set of genes expressed in xylem across all individuals, and found several instances of alternative splicing, particularly in cell wall-related genes and that these alternative splicing events differed significantly across individuals.

An increasingly common study design is the construction of a gene expression atlas for a species, which involves determining the expression level of every gene in the genome in various different tissues and/or conditions. Gene expression atlas studies have been performed in various plant species (see, for example, **Table 2**), and several expression atlas datasets are available on Phytozome (phytozome.jgi.doe.gov). The *P. trichocarpa* RNA-Seq gene expression atlas consists of genome-wide gene expression measurements across several different samples of tissue and condition combinations, including root, root tip, stem, node, internode, bud, leaf, and flower tissues. Root and stem tissues included several samples varied by nitrogen source. Bud, leaf, and male and female flowers included several samples of different stages of maturity. Gene expression values for 40 of these samples are currently publicly available in PhytoMine on the Phytozome web interface (Goodstein et al., 2012; https://phytozome.jgi.doe.gov/index.html). To our knowledge, this is the largest RNA-Seq expression study performed in *Populus*.

**Table 2:** Examples of gene expression atlas studies in plants.

Species	Reference	Samples	Method
*Arabidopsis thaliana*	Schmid et al. (2005)	79 samples from various tissues and developmental stages	Affymetrix GeneChip
*Sorghum bicolor*	McCormick et al. (2018)	47 combinations of tissues (roots, leaves, stems, panicles) and developmental stages (juvenile, vegetative, reproductive)	RNASeq
*Glycine max*	Severin et al. (2010)	14 tissues from different developmental stages	RNASeq
*Lotus japonicus*	Verdier et al. (2013)	237 samples of 8 tissues across various conditions	Affymetrix GeneChip
*Medicago truncatula*	Benedito et al. (2008)	18 samples from tissues across different developmental stages	Affymetrix GeneChip
*Barley*	Druka et al. (2006)	15 tissues identified from eight developmental stages	Affymetrix GeneChip
*Rice*	Wang et al. (2010)	31 tissues spanning life cycle of rice plant for 2 rice varieties, 8 samples from stages in the tissue culture process	Affymetrix GeneChip
*Panicum virgatum L (Switchgrass)*	Zhang et al. (2013)	Tissues (roots, shoots, and panicle) and developmental stages (leaf development, stem elongation and reproduction)	ESTs
*Vitis vinifera*	Fasoli et al. (2012)	54 samples from tissues spanning different developmental stages	NimbleGen microarray and RNASeq

### Metabolomics

Metabolomics studies involve measuring the quantities of metabolites within a sample. While targeted metabolomics studies aim to only measure and identify a select few metabolites within a sample (for instance using standards), untargeted metabolomics involves the measuring as many metabolites as possible within a sample (Patti et al., 2012). Identification of metabolites in untargeted metabolomics studies is much more challenging than that of targeted metabolomics studies. While the candidate identities of many metabolite peaks can be determined through database matching or manual inspection of mass spectra with the necessary expertise, many metabolites will remain unidentified or partially identified.

Several targeted and untargeted metabolomics studies have been performed in *Populus*. In a study by Morreel et al. (2006), metabolite levels of 15 flavonoids were measured using high-performance liquid chromatography (HPLC), and subsequently mQTL (metabolite quantitative trait loci) based on amplified fragment length polymorphisms (AFLPs) was used to identify potential genes involved in rate-limiting steps of flavonoid biosynthesis. Kaling et al. (2015) performed untargeted metabolomics on UV-B treated vs. control *P. alba* × *P. tremula* plants using Fourier transform ion cyclotron resonance mass spectrometry (FT-ICR-MS). This allowed for the investigation of the effect of UV radiation on the metabolome. Tuskan et al. (2012) performed gas chromatography–mass spectrometry (GC/MS) analysis of 16 individual trees in *P. deltoides* and *P. nigra*, and showed gender-specific accumulations of metabolites in floral buds. In Hamanishi et al. (2015), transcriptomic and metabolomic data of six *P. balsamifera* were collected using Affymetrix microarrays and GC/MS, respectively, to investigate the response of the metabolome and transcriptome to drought stress. Tschaplinski et al. (2014) used GC/MS-based metabolomics on samples of *P. trichocarpa* and *P. deltoides* roots colonized with *Laccaria bicolor* as well as control samples to investigate the different metabolic responses to colonization. One interesting result was that increased levels of defense-related compounds were found in the incompatible host, *P. deltoides*, whereas some defense compounds were significantly lower in the compatible host, *P. trichocarpa*. A recent study by Veach et al. (2018) investigated the effects on the metabolome of *P. deltoides* when downregulating *PdKOR1*, a glycosyl hydrolase gene involved in cellulose biosynthesis. GC/MS analysis of root tissue from *PdKOR1* RNAi lines vs. control lines showed that caffeic acid derivatives, metabolites involved in fatty acid metabolism as well as salicylates and flavonoids were upregulated in RNAi lines when compared to control lines Veach et al. (2018). Additionally, Tschaplinski et al. (2019) reported the differential foliar metabolomic responses of *P. deltoides* responding to acute vs. chronic drought, with the former inducing the largest osmotic adjustment (1.42×), with the greatest accumulations in the large, complex higher-order salicylate conjugates, and hydroxycinnamic acid conjugates of salicin; the populosides were particularly elevated.

A GWAS using SNPs from 917 *P. trichocarpa* accessions as well as GC/MS-based metabolomics and RNA-Seq-based gene expression measurement identified hydroxycinnamoyl- CoA:shikimate hydroxycinnamoyl transferase 2 (PtHCT2) as a gene that is significantly associated with the levels of 3-O-caffeoylquinic acid, and also identified transcription factors that regulate this gene (Zhang et al., 2018).

### Proteomics

Proteomics involves identifying and quantifying the levels of the protein component of cells within a sample. This is an important layer of data to consider, as it allows for the investigation of the cellular components that participate directly in metabolic pathways, cell structure, and development (Chen and Harmon, 2006). The relationship between protein levels and transcript levels varies depending on the scenario. For example, as reviewed by Liu et al. (2016b), while “at steady state, mRNA levels primarily explain protein levels,” this relationship can change when measuring transcript/protein levels after a state transition, as there is an expected delay between mRNA and protein synthesis (Liu et al., 2016b). The relationship between mRNA and protein levels is thus complicated, and we refer the readers to Liu et al. (2016b) for a detailed review on the dependencies between these two ‘omics layers. It is important to note that separate identification and quantification of proteins is important to fully understand this ‘omics layer, including the set of post-translational modifications undetectable through other ‘omics layers.

Several proteomics studies have been performed in *Populus* investigating changes in the proteome due to different developmental stages or conditions. There is a particular abundance of studies investigating the response of the *Populus* proteome to drought stress. Zhang et al. (2010) investigated the sex-related differences proteomic response to drought stress in *P. cathayana* and found significant sex-dependent responses to drought stress, particularly in chloroplast-related processes such as the Calvin cycle, electron transport chains, and chloroplast components. They also found that the growth rates of male trees were less affected by drought than females, and that chloroplasts were less damaged by drought in males than females (Zhang et al., 2010). Durand et al. (2011) performed a proteomic study to investigate the different drought responses of different tissues in *Populus tremula L*. × *P. alba L*., illustrating how some tissues are affected sooner by drought than others. Abraham et al. (2018) investigated the proteome of *P. deltoides* in response to different types of drought stress. Two separate drought treatments were used: a cyclic drought treatment and a prolonged, “slow-drying” water deficit. Differentially abundant proteins were determined for each of the two drought treatments, and, interestingly, these sets of differentially abundant overlapped by only around 10%. This study illustrated diversity in responses to different types of water deficit stress.

Several studies have also investigated proteome profiles at different stages of development in *Populus* species (Liu et al., 2015; Obudulu et al., 2016). Proteomics is thus an important data layer that can provide information about cellular function and responses that could not be gained by other ‘omics layers. As the “end point” of the central dogma, proteins are the biomolecules that provide a large part of the functional capacity of a cell, and are a crucial aspect of understanding the functioning of the system.

### Epigenomics

#### DNA Methylation

Epigenetics involves the study of additions of chemical groups to chromatin (either the DNA or histones) that do not change the underlying DNA sequence. These modifications consist of histone methylation (Liu et al., 2010), histone acetylation (Lusser et al., 2001), and DNA methylation (Finnegan et al., 1998). Histone methylation occurs on lysine and arginine residues in histones and can have a silencing or activating effect on gene expression, depending on which lysine residue is methylated (Liu et al., 2010). Histone acetylation involves the addition of an acetyl group to the ϵ amino group of lysine residues in the N-terminal tails of histones that protrude from the histone octamer complex (Lusser et al., 2001). While histones are usually positively charged, and DNA is negatively charged, acetylation can neutralize the positive charge of the histones, resulting in a weaker association between the DNA and the histone complex. This can allow for greater access for transcription factors to the DNA and can thus impact gene expression (Lusser et al., 2001). DNA methylation involves the addition of a methyl group to cytosine residues (Law and Jacobsen, 2010). This is known to have a gene-silencing affect. DNA methylation in plants occurs mostly in repetitive DNA and TEs. This is thought to be a protective mechanism to silence transposons. DNA methylation is also found within the transcribed regions of genes in plants (Suzuki and Bird, 2008). This gene body methylation does not have a silencing effect like promotor methylation does, but appears to lead to stable gene expression across many tissues (Zilberman et al., 2007;Suzuki and Bird, 2008). Epigenetic modifications can be inherited from parents or occur as a result of a stress response (Chinnusamy and Zhu, 2009; Lämke and Bäurle, 2017).

Two major whole-genome sequencing-based approaches for determining DNA methylation across a genome are methyl-DNA immunoprecipitation (MeDIP) followed by sequencing (MeDIP-Seq) (Jacinto et al., 2008) or treatment of DNA with bisulfite followed by sequencing (Frommer et al., 1992; Krueger et al., 2012). MeDIP-Seq involves shearing of DNA into small fragments of 300–600 bp and subsequent immunoprecipitation of methylated DNA using an antibody raised against 5-methylcytidine. The resulting immunoprecipitated fragments are sequenced, and mapping of the reads to the reference genome reveals the regions of the genome that contain methylated cytosines. It is important to note that the resolution of the methylation results cannot exceed the fragment size. In bisulfite sequencing, DNA is pre-treated with sodium bisulfite, which converts un-methylated cytosine residues to uracil residues, while methylated cytosine residues remain unchanged. Subsequent sequencing provides single-base resolution of methylated cytosines (Frommer et al., 1992; Krueger et al., 2012). See published papers of Laird (2010) and Bock (2012) for useful reviews on DNA methylation analysis.

Vining et al. (2012) investigated DNA cytosine methylation in seven different tissues in *P. trichocarpa*, including bud, male catkin, female catkin, leaf, root, xylem, and phloem. DNA methylation was determined using MeDIP-Seq followed by mapping of reads to the *P. trichocarpa* reference genome. Reads mapped most frequently to intergenic regions and repeat sequences, although promotor methylation and gene body methylation were observed. Variation in methylation across tissues was observed at certain chromosomal locations. A surprising result was that gene body methylation appeared to be a stronger repressor of transcription than promotor methylation (Vining et al., 2012). Slavov et al. (2012) made use of the methylation data generated by Vining et al. (2012) in investigating the correlates of recombination in the *P. trichocarpa* genome and found that DNA within recombination hotspots were significantly less methylated than non-hotspots.

A follow-up study by Vining et al. (2013) used MeDIP-Seq to examine methylation levels in another three tissues, focusing on regeneration and de-differentiation tissue types, namely, internode stem from propagated explants, callus, and internodes from regenerated plants. The MeDIP-Seq reads for the 10 different *P. trichocarpa* tissues from these two studies have been mapped to the version 3.2 genome assembly and are available on Phytozome (Goodstein et al., 2012).

A stress response methylome study was performed in *P. trichocarpa* in which DNA methylation was measured in drought stress and control plants using bisulfite sequencing (Liang et al., 2014). The number of methylated cytosines increased significantly under drought stress and the genes differentially methylated in drought stress vs control plants were enriched for regulatory GO terms. This study also performed the first investigation of alternative splicing in *P. trichocarpa* and identified multiple forms of alternative splicing. An interesting finding was also that all fusion genes identified were methylated (Liang et al., 2014).

Lafon-Placette et al. (2013) investigated the component of the *P. trichocarpa* methylome in open chromatin by isolating chromatin sensitive to DNase I and performing MeDIP-Seq on the resulting DNA. Extensive gene body methylation was found, more so than was originally reported for *P. trichocarpa*.

The two studies by Vining et al. (2012, 2013) provide the best available methylation dataset to use as an association network layer in *P. trichocarpa* as it covers the broadest range of sample types.

#### AtAC-Seq

The assay of transposase-accessible chromatin (ATAC-Seq) uses a transposase to insert sequencing adaptors into accessible regions of chromatin (the areas in between nucleosomes) (Buenrostro et al., 2013, Buenrostro et al., 2015). The resulting fragments are then PCR-amplified and sequenced. This results in nucleotide resolution of open chromatin. There were challenges in applying this method to plant cells due to contaminating DNA from chloroplasts and mitochondria because chloroplast and mitochondria genomes are very accessible to the transposase and thus lower the efficiency of the technique. Lu et al. (2016) developed a technique, fluorescence-activated nuclei sorting (FANS)-ATAC-Seq, which involves sorting of nuclei using flow cytometry prior to ATAC-Seq analysis. Bajic et al. (2018) describe protocols for the isolation of plant nuclei from different cell types for further analysis using ATAC-Seq. A recent study by Maher et al. (2018) applied ATAC-Seq to *A. thaliana*, *M. truncatula*, *Solanum lycopersicum* (tomato), and *Oryza sativa* (rice). An interesting finding was that in all four species, most open chromatin sites were in non-transcribed regions.

ATAC-Seq is a relatively new technology, and, to date, no study has been published on the application of ATAC-Seq in *P. trichocarpa*.

### DAP-Seq

DNA affinity purification sequencing (DAP-seq) is a technique used to determine transcription factor binding sites (O’Malley et al., 2016). This technique involves coupling a particular transcription factor of interest to affinity beads. Fragmented genomic DNA is eluted over the beads, retaining only DNA fragments that bind to the transcription factor. Subsequently, the retained fragments are sequenced. The first study describing this technique demonstrated its use in identifying the Arabidopsis “cistrome”—the binding location/motifs of 1,812 transcription factors (O’Malley et al., 2016). The DAP-seq protocol was published by Bartlett et al. (2017).

To date, no DAP-seq study has been performed in *P. trichocarpa*. This would be an incredibly valuable data layer to investigate the transcription factor regulatory network of *P. trichocarpa*.

## Data Integration

### Multi-Omic Studies and Data Integration

The current era has an extensive suite of technologies capable of measuring and characterizing several aspects of a cellular system, such as NGS technologies for genomics, transcriptomics, and epigenomics as well as metabolomics and other phenotypes. An untargeted approach is often favored over a targeted approach as this attempts to capture information about the entire system and understand the organism as a whole. In the review by Weckwerth (2011), it is highlighted that the next step in understanding complex systems will involve the integration of these different data layers. An important and challenging task that data integration can help solve is the identification of new candidate genes involved in complex phenotypes (Hassani-Pak and Rawlings, 2017; Valledor et al., 2018), which can then be validated using genetic/molecular biology tools. It is particularly difficult to generate hypotheses that suggest the mechanism of a gene’s effect on a particular phenotype. Prioritizing candidate genes and hypothesizing the mechanism of the effect requires multiple data types, such as gene–phenotype associations, expression/co-expression information, knowledge from literature, annotation information, protein–protein/protein–DNA interactions, and epigenetic modifications, to name a few (Hassani-Pak and Rawlings, 2017). This presents a challenge because of the heterogeneous nature of these data types, and the fact that they are often distributed across different databases and represented as different structures (Hassani-Pak and Rawlings, 2017). There is thus an increasing value in databases that integrate various layers of data from various sources (Hu et al., 2018), for example, Knetminer (Hassani-Pak et al., 2016; Hassani-Pak, 2017) and String (Mering et al., 2003; Szklarczyk et al., 2010; Fukushima and Kusano, 2014;Szklarczyk et al., 2017).

Data integration requires that the various data layers be coerced into a uniform data structure. The data collected from various techniques can each be represented as a matrix/table of samples and variables, as illustrated in the review by Weckwerth (2011). Once represented as a matrix, there are various data structures/analysis approaches that can be used to integrate and analyze the data. This can range from multivariate analysis such as Orthogonal Projections to Latent Structures (OPLS) (Bylesjö et al., 2007) to networks (Krishnan et al., 2016; Hassani-Pak and Rawlings, 2017) and signal processing, such as that seen in the study by Spencer et al. (2006).

There are two main data integration strategies that have been applied in poplar, namely, network-based data integration and signal-based integration. While there are many useful databases that present poplar gene networks or different sources of ‘omics data [see, for example, “GFDP: the gene family database in poplar” (Wang et al., 2018a) and also the useful review by Lee et al. (2015)], we will focus below on strategies that have computationally integrated several different ‘omics data layers. This section describes the theory behind integration strategies and data structures/approaches applied in poplar, such as networks and signal processing. Examples of the various ‘omics layers in these data structures are then presented. Thereafter, examples in which these data structures/methods are used in the analysis of multiple biological data types are discussed, focusing on examples in *Populus* species.

### Networks

#### Network Theory

**Figure 1:**
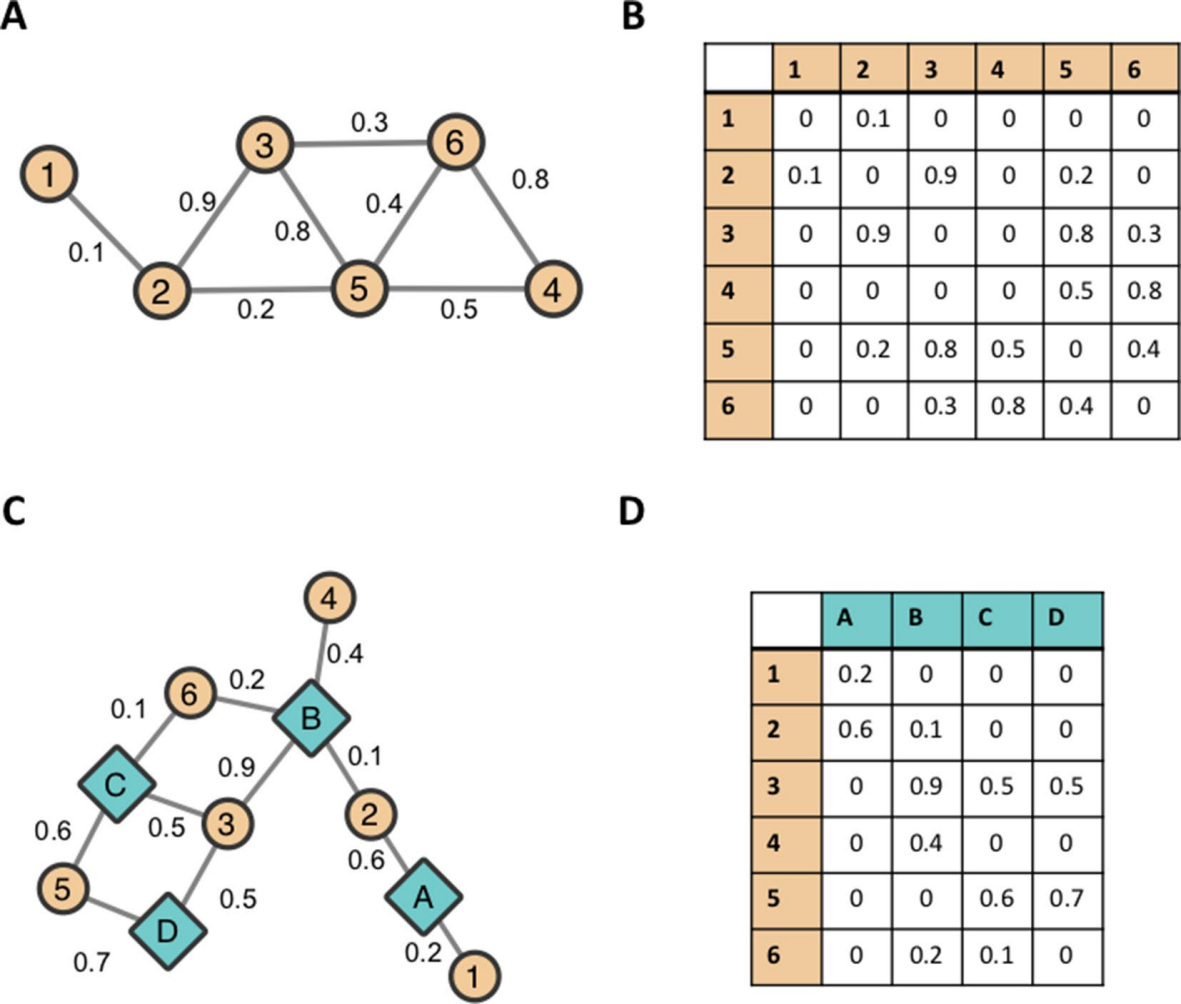
Example networks. A small, standard undirected network represented **(A)** visually as a collection of nodes and edges and **(B)** as an adjacency matrix. A bipartite network represented **(C)** visually and **(D)** as an adjacency matrix.

Networks are useful mathematical structures that represent a system in terms of its components, and pairwise interactions between the components (Barabasi and Oltvai, 2004). The field of Network Theory has its origins in Graph Theory. Intuitively, a graph (or network) is a set of objects (nodes) connected by lines (edges) as shown in **Figure 1A**. In biological network applications, nodes represent a biological object of interest and edges represent associations/interactions/similarities between these biological objects.

A graph can be represented numerically as a matrix, namely, an Adjacency Matrix (Golumbic, 2004). The Adjacency Matrix associated with the small example graph in **Figure 1A** is shown in **Figure 1B**. Each edge *e_ij_* in a graph can be assigned a real number weight *w_ij_* that represents the strength of the relationship between the two nodes it connects. A weighted graph can be mathematically represented as a Weighted Adjacency Matrix. This matrix is constructed in a similar manner to the normal Adjacency Matrix.

A bipartite graph involves nodes that can be partitioned into two non-overlapping sets. Intuitively, this means that a bipartite graph (or a bipartite network) consists of two classes of nodes in which nodes of one class can only be connected to nodes of the other class. An example of a bipartite network is shown in **Figure 1C**, and its matrix representation is shown in **Figure 1D**. Mathematical definitions of networks and adjacency matrices can be found in **Supplementary Text 2**.

Networks are useful tools for modeling and analyzing complex biological systems by representing biological molecules/components as nodes (e.g. genes, proteins or metabolites) and representing the relationships/interactions/similarities between them as edges (Barabasi and Oltvai, 2004). For example, networks can model co-expression relationships between genes, sequence similarity between genes, physical interactions between proteins, or correlations between metabolites. Bipartite networks are particularly useful when representing relationships between different types of biological objects/concepts. For example, Goh et al. (2007) use bipartite networks to represent the human “diseaseome,” connecting human diseases to their associated genes. Also, Weighill et al. (2019a) used bipartite network representations of GWAS results in order to characterize potentially pleiotropic associations in *P. trichocarpa*. As discussed in Weighill et al. (2019a), bipartite networks are useful structures for the representation and visualization of high-dimensional data. One set of nodes in a bipartite network can represent the variables (axes) of a space, while the other set of nodes represents the points within that space (samples). This representation allows for high-dimensional datasets to be visualized in two dimensions as a network. Networks allow for biological datasets to be visualized in an intuitive manner and network visualization packages such as Cytoscape (Shannon et al., 2003) provide an interactive environment for network visualization. However, networks are not simply useful as a visualization tool. Networks provide a data structure that can be computed upon, allowing further analysis to be performed on a dataset represented as a network. Examples of such analysis methods include network-based clustering algorithms such as Markov Clustering (MCL) (Enright et al., 2002) and Weighted Gene Co-expression Network Analysis (WGCNA) (Zhang and Horvath, 2005) that cluster the nodes of a network into groups based on the topology of the underlying network. Datasets represented as networks are also very easily merged with each other. This feature makes networks a useful tool for combining information from different data sources to create an integrated and holistic environment for data interpretation.

#### GWAS Networks

Network approaches have been applied to GWAS analyses in order to interpret or further analyze the resulting lists of SNPs and *p* values. These often involve mapping the resulting SNPs associated with phenotypes to their respective genes, and then projecting these genes into protein–protein interaction networks (Akula et al., 2011) or co-expression networks (Farber, 2013) in order to identify other putative causal genes, or to form sets or subnetworks of genes putatively affecting the same phenotype (Leiserson et al., 2013).

The results of a GWAS can be viewed as a bipartite network where the set of nodes can be partitioned into a set of SNPs and a set of measured phenotypes, and the edges connect SNP nodes to phenotype nodes they are significantly associated with. SNPs can be connected to multiple phenotypes, and phenotypes can be connected to multiple SNPs. A toy example of such a network can be seen in **Figure 1C**. Teal, diamond-shaped nodes represent measured phenotypes A–D and orange, circular nodes represent SNPs 1–6. Each edge represents GWAS associations between SNPs and phenotypes. This representation of GWAS results has been used to estimate pleiotropy within a Human-Phenotype Network, calculated as the average degree of the gene nodes within a gene–phenotype bipartite network (Darabos et al., 2014). Another example can be seen in a study by Fagny et al. (2017) in which the results of an expression quantitative trait loci (eQTL) were represented as a bipartite network, connecting SNPs to genes if the expression level of the gene was significantly associated with the SNP (Fagny et al., 2017). Recently, Weighill et al. (2019a) presented a method for characterizing multi-phenotype association signatures from GWAS results in order to investigate potentially pleiotropic interactions between genes and phenotypes. This method involves a decomposition relationship between three GWAS-derived bipartite networks, which allow for detailed pleiotropic signatures to be characterized, and, furthermore, allows for genes to be clustered based on the detailed topology of SNP–phenotype associations within the gene. This method was demonstrated on metabolomic GWAS results in *P. trichocarpa* and suggests applications of this method in identifying promising target genes of interest to modification or selective breeding (Weighill et al., 2019a).

#### Co-Expression, Co-Methylation, and Correlation Networks

**Figure 2:**
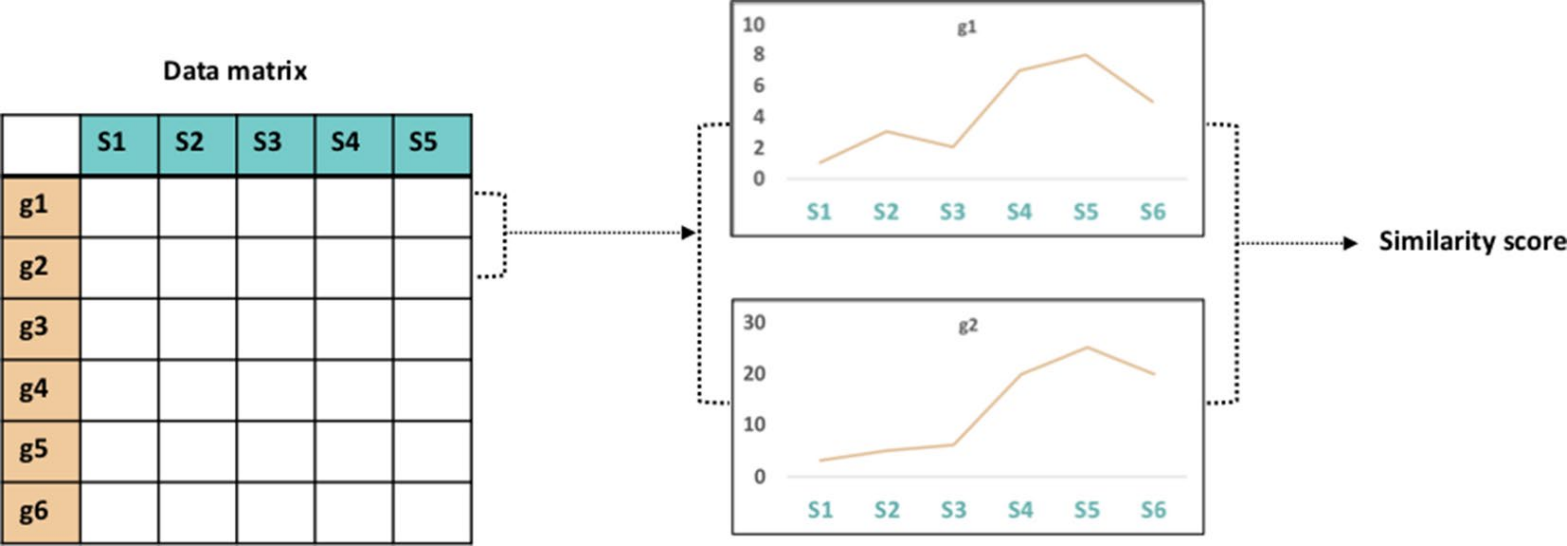
Vector similarity. Gene association network comparison involves construction of a data matrix of measurements (e.g., gene expression) for all genes in a genome across various samples. Calculation of the similarity between all pairs of gene vectors results in a similarity score.

Several of the ‘omics data layers discussed in the section *Sources of ‘Omics Data Layers* can be used to construct gene networks, such as gene co-expression networks and gene co-methylation networks. These networks require some quantity, such as gene expression, to be measured for every gene across multiple samples representing different conditions, tissues, or perturbations. A common way to construct gene association networks is to calculate the similarity between the profiles of all pairs of genes (**Figure 2**) and then apply a threshold [for examples, see Li et al. (2015) and Weighill and Jacobson (2017)]. The choice of similarity metric can have a large impact on the resulting network topology, as shown in a study by Weighill and Jacobson (2017).

Co-expression networks have been used for various applications, including gene function investigations, gene module and regulatory hub gene investigations, as well as comparative co-expression network analysis across different species (Aoki et al., 2007; Li et al., 2015; Serin et al., 2016; Emamjomeh et al., 2017;Schaefer et al., 2017). Movahedi et al. (2012) described an approach for incorporating gene homology information in order to compare gene co-expression modules across plant species to identify clusters that are conserved across species. The overall functional impact of modules of sets of co-expressed genes can be investigated using enrichment of functional ontologies such as GO (Gene Ontology Consortium, 2004) and MapMan (Thimm et al., 2004) [see, for example, Emamjomeh et al. (2017)].

Horvath and Dong (2008) presented a useful set of network topology measures to characterize the structure of a co-expression network (or any network). These measures ranged from global network measures such as centralization, density, and heterogeneity to node-based metrics such as connectivity and the clustering coefficient. Yip and Horvath (2007) also developed a new network measure/transformation called Topological Overlap, which calculates the “connectedness” of two nodes based on direct connections as well as indirect connections *via* their neighbors. This provides an extra transformation that can be performed on a similarity network, which considers not only the similarity between expression profiles of two genes in question but also the expression profiles of their network neighbors, and thus can help address the problem arbitrary thresholds missing important edge connections. This Topological Overlap measure is an integral part of a popular gene co-expression pipeline called WGCNA developed by Langfelder and Horvath (2008). An extension of the Topological Overlap measure, called Cross-Network Topological Overlap, was developed by Weighill and Jacobson (2017), which can be used to compare the similarity in the neighborhoods of a given node in two distinct networks.

Several studies in *Populus* species have involved co-expression networks, some focusing on co-expression networks as the main aspect of the investigation, and others using co-expression networks as a supplementary investigation surrounding the functions of a specific set of genes. Netotea et al. (2014) investigated differences in the genome-wide co-expression networks of *P. trichocarpa*, *O. sativa*, and *A. thaliana* constructed from publicly available expression data. It was found that while individual gene–gene co-expression relationships were different between the three species, overall neighborhoods of genes were significantly conserved across species. Another interesting finding was that orthologs with the most sequence similarity did not have the most similar expression pattern [“expressolog,” as defined in Patel et al. (2012)].

An interesting co-expression study by Grönlund et al. (2009) constructed co-expression networks from 1024 publicly available microarray datasets for *P. tremuloides* by jack-knife re-sampling half of the number of samples 100 times, calculating the Pearson correlation between all pairs of genes in each jack-knife re-sample, converting the Pearson correlations to distance metrics, and subsequently constructing 100 minimum spanning trees (MSTs) and merging the resulting networks. This approach of re-sampling allowed for the identification of rarer interactions between genes that would not have been identified through only looking at the dataset as a whole. Another whole genome co-expression study in *Populus* was performed by Ogata et al. (2009) in which 95 publicly available *P. trichocarpa* microarray expression datasets were used to construct a co-expression network and extracted co-expression modules, which were released in a publicly available database.

Several studies of specific genes in *Populus* incorporated co-expression elements into their analysis. Tian et al. (2017) investigated the role of *P. trichocarpa* Na+/H+ antiporters in stress responses, as well as potential functional divergences within the family of these NHX genes. Using a co-expression network from publicly available data on Phytozome, they showed divergence in the expression pattern of members of this family. Several studies in *Populus* performed WGCNA of genes responding to certain stresses/conditions, including control vs drought conditions (Xue et al., 2016) in *P. tremula* × *alba*, controls vs jasmonic acid, and salicylic acid treatments in a *P. deltoides* × *P. euramericana* hybrid (Luo et al., 2019), and also a developmental gradient of stem tissue (Chao et al., 2019). In a characterization of DWARF14 genes in *P. trichocarpa*, co-expression networks showed divergent expression between the two DWARF14 (Zheng et al., 2016). In another recent study by Tuskan et al. (2018), genes having a GWAS association with callus formation were identified, and the co-expression patterns of these genes were investigated using a co-expression network constructed from the *P. trichocarpa* gene expression atlas, and identified interesting clusters of positive and negative co-expression relationships between these genes, showing a clear regulatory pattern. It is evident that co-expression networks are a well-developed and widely used data layer in various organisms including *Populus*.

Co-methylation networks are a newer approach looking at the similarity between the methylation patterns of genes, and a more limited number of studies using co-methylation networks were found. However, they are a valid and useful data layer that carries information not present in co-expression datasets.

In a study by van Eijk et al. (2012), methylation and gene expression data were collected for several human individuals to investigate the relationship between these two data layers. WGCNA was used to construct co-expression and co-methylation networks, and subsequently to identify co-expression and co-methylation modules. In general, co-expression and co-methylation modules had very few overlapping genes, although both co-expression and co-methylation modules showed significant functional enrichment for various GO terms. Linear regression was also used to identify relationships between methylation and expression across individuals in which both positive and negative relationships were identified (van Eijk et al., 2012). Various other co-methylation network analyses have been performed in human cancer investigations (Akulenko and Helms, 2013; Bartlett et al., 2014;Ha et al., 2015).

#### SNP Correlation

SNP correlation networks involve calculating the correlation/co-occurrence between SNPs across a population, and can be converted to gene–gene networks by mapping SNPs to the genes in which they reside. The Custom Correlation Coefficient (CCC) is an allele-specific correlation metric proven to be useful in identifying sets of SNPs (“blocs”) that can be tested against complex phenotypes to uncover combinatorial genetic associations that affect the phenotype (Climer et al., 2014a;Climer et al., 2014b). The edges in the SNP correlation network can also be interpreted as potential co-evolutionary relationships, particularly when the variants in question reside on different chromosomes. The CCC is defined for bi-allelic SNPs. For a given pair of sites *i* and *j*, the CCC is calculated four times, once for each pair of alleles *x* and *y* between the two sites. The CCC between alleles *x* and *y* at positions *i* and *j*, respectively, is defined as:

(1) $${\mathrm{CCC}}_{i_{x}j_{y}}=\frac{9}{2}R_{i_{x}j_{y}}\left(1-\frac{1}{f_{i_{x}}}\right)\left(1-\frac{1}{f_{j_{y}}}\right)$$

where $R_{i_{x}j_{y}}$ represents the relative co-occurrence of *x* and *y* at positions *i* and *j*, $f_{i_{x}}$ represents the frequency of allele *x* at position *i*, and $f_{j_{y}}$ represents the frequency of allele *y* at position *j* (Climer et al., 2014a;Climer et al., 2014b).

CCC has been used to investigate the genetic underpinnings of heart disease (Climer et al., 2014b), psoriasis (Climer et al., 2014a), and genes implicated in various other diseases (Climer et al., 2015). This metric was also applied in a study by Bryan et al. (2018) in *P. trichocarpa*. The positioning of the two DWARF14 paralogs in the *P. trichocarpa* SNP correlation network was investigated, indicating that they appeared to have different co-evolution partners, potentially indicating functional divergence (Bryan et al., 2018). The CCC metric has been ported to run on graphics processing units (GPUs) providing a significant increase in speed (Joubert et al., 2018; Joubert et al., 2019).

Kogelman and Kadarmideen (2014) constructed SNP correlation modules by calculating the Pearson correlation between pairs of variants across individuals followed by topological overlap clustering using WGCNA. This method was termed “WISH” (Weighted Interaction SNP Hub) and was considered an extension of WGCNA to genotype data. Later, in 2017, the developers of WGCNA published an extension of the method to construct SNP correlation networks from GWAS associations, termed “WSCNA” (Weighted SNP Correlation Network Analysis), which involves clustering SNPs based on beta coefficients from a GWAS analysis (Levine et al., 2017), and describe the use of these networks in calculating polygenic risk scores.

#### Network-Based Data Integration

A useful review by Gligorijević and Pržulj (2015) classifies network-based data integration into two categories, namely, homogeneous and heterogeneous integration. Homogeneous integration involves integrating networks with the same type of nodes, but different edge types, for example, a gene co-expression network and a gene interaction network. Heterogeneous data integration involves integrating networks with both different node types and edge types. These strategies for data integration are then subdivided into groups based on the stage at which data integration occurs. Early integration involves integration of the datasets, and a single model is built on a combined dataset. This appears similar to the definition of *Concatenation-based integration* as described by Ritchie et al. (2015). Late integration involves building separate models from each individual dataset and subsequently combines the information in the separate models. This is similar to *Model-based integration* as described by Ritchie et al. (2015). A third integration strategy described by Ritchie et al. (2015), *transformation-based integration*, involves transforming multiple datasets into an intermediate, common structure, such as a network, which are then merged before the constructing further models.

Two of the most exhaustive network-based data integration tools are String (Search Tool for the Retrieval of Interacting Genes) and KnetMiner, both of which are online, freely accessible resources. STRING is an online, publicly available database of protein interactions, incorporating various data types and data sources, including co-expression, co-occurrence, physical interactions, sequence homology, and associations from textmining (Mering et al., 2003; Szklarczyk et al., 2010; Szklarczyk et al., 2017). The user can search for genes and resulting network neighborhoods can also be clustered using K-means clustering and MCL (Enright et al., 2002). Protein 3D structure as well as functional enrichment information is also displayed. The STRING database can also be queried through the Cytoscape network visualization app (Shannon et al., 2003; Szklarczyk et al., 2017). Certain sets of publicly available data for *P. trichocarpa* are available in STRING. KnetMiner is a publicly available tool/database consisting of heterogeneous “knowledge” networks for 11 species, including *P. trichocarpa*, and includes layers of information of different types and sources represented as networks, such as GWAS data, sequence homology relationships, annotation information, metabolic pathways, protein interactions, and occurrence in scientific literature (Hassani-Pak et al., 2016; Hassani-Pak, 2017). KnetMiner allows the user to search not only for genes, but also for concepts, phenotypes, or pathways. A score (KNETscore) is then calculated to rank genes based on their relevance of the neighborhood to the search terms. KnetMiner provides useful network visualizations as well as a chromosomal view indicating the location on the chromosomes in which the genes occur and an “evidence view” indicating the number of nodes/concepts of different types in the neighborhood of the genes in question.

The Mergeomics R package and webserver allows one to integrate GWAS summary statistics with other biological pathways and gene networks, and perform enrichment analyses as well as Weighted Key Driver Analysis (Arneson et al., 2016). This involves identifying hub genes in a selected/uploaded gene network, and subsequently overlaying phenotype-associated genes from uploaded GWAS analyses, and reports key drivers for each of these genes (Arneson et al., 2016). Key drivers and their neighborhoods can then be visualized using Cytoscape Web.

Mizrachi et al. (2017) developed an interesting network-based data integration approach to combine pathway information from KEGG, eQTL associations, and gene expression data in *Eucalyptus*. The network-based integration approach involves constructing a gene interaction network based on information in KEGG as well as eQTL associations with biomass and wood traits. The adjacency matrix of this network is then multiplied with a gene expression matrix, which results in a “network-diffused gene expression” matrix. This adjusts gene expression values based on those of neighboring genes in the gene interaction network. These new gene expression profiles are then correlated with each trait to identify genes of relevance to wood properties and biomass (Mizrachi et al., 2017).

Walley et al. (2016) performed a study that compared the topologies of various gene association networks in Maize. A gene co-expression network, a protein-co-expression network, and a phosphoprotein co-expression network were constructed and clustered into modules using WGCNA, and the edge conservation between the networks was calculated using the Jaccard index, and found that 6.1% of the edges were shared between the protein co-expression and gene co-expression networks. Functional enrichment using MapMan (Thimm et al., 2004) terms was performed on modules of co-expressed genes/proteins from the two networks, and similar enriched functions were found in both networks.

NetICS (Network-based Integration of Multi-omics Data) is a data integration strategy based on graph diffusion (Dimitrakopoulos et al., 2018). This method was developed by Dimitrakopoulos et al. (2018) in order to prioritize cancer genes. A directed gene interaction network was constructed from publicly available data that included multiple types of relationships, including phosphorylation, co-expression, activation, and inhibition. Aberrant genes (i.e., those found to be differentially impacted in a case/control experiment) are marked and network diffusion is used to predict “mediator genes” that link upstream “genetically aberrant” genes to downstream gene expression changes (Dimitrakopoulos et al., 2018). This approach successfully identified many known cancer genes.

Gutiérrez et al. (2007) investigated gene expression in *A. thaliana* under carbon and nitrogen treatments. A separate gene interaction network was constructed using publicly available protein–protein and protein–DNA interactions, as well as miRNA–RNA interactions and the *Arabidopsis* metabolic pathway. A subnetwork consisting of C/N responsive genes and their neighbors in the multi-network was constructed. Clustering of this subnetwork revealed interesting regulatory subnetworks.

Bunyavanich et al. (2014) used a multi-omic network-based approach to investigate allergic rhinitis. GWAS was performed on 5633 genotyped individuals, and gene expression was measured in 200 of these individuals. Gene co-expression network and modules were constructed using WGCNA. Co-expression modules that contained genes that harbored or were near to GWAS-associated genes were considered candidate modules associated with allergic rhinitis. Associations between SNPs and gene expression were determined (called “eSNPs”), which are SNPs within 1 MB of a gene, which is also associated with the expression of the gene. Modules enriched in eSNPs were also identified, and it was found that the candidate allergic rhinitis modules were enriched in eSNPs associated with allergic rhinitis, and mitochondrial pathways were identified as important components of allergic rhinitis using functional enrichment (Bunyavanich et al., 2014).

Calabrese et al. (2017) integrated GWAS and co-expression data in an investigation into genes affecting bone mineral density. Genes identified as associated with bone mineral density in a GWAS analysis were mapped onto a co-expression network, which was subsequently clustered into modules. Co-expression modules that were enriched for GWAS hits were then identified.

Liu et al. (2016a) constructed a “co-functional” gene network for *P. trichocarpa*, making use of multiple data sources, including genomics data, poplar gene expression data from microarray experiments, as well as various sources of annotation including PFAM, GO, KEGG pathways, MapMan annotations, and MetaCyc. The co-functional network is accessible through the PoplarGene webserver, which also contains tools for gene prioritization (Liu et al., 2016a).

Weighill et al. (2018) presented a “Lines of Evidence” (LOE) approach for integrating data and identifying new candidate genes involved in a function of interest. The LOE approach takes as input a set of anchor genes/phenotypes thought to be involved in a given function of interest based on annotation and literature/expert knowledge. Thereafter, a LOE score is calculated for every gene in the genome, quantifying its connectivity to the input anchor genes across various ‘omics network layers. These scores then allow genes to be ranked based on how much evidence exists connecting them to a given function across several data layers. This method, demonstrated and applied in *P. trichocarpa*, integrated several layers of association networks, including a gene co-expression network, gene co-methylation network, gene co-evolution network, as well as two GWAS networks and identified new promising candidate genes potentially involved in lignin biosynthesis and regulation (Weighill et al., 2018). The association networks constructed in this study were also used to provide context to candidate genes identified in a multi-trait GWAS analysis in *P. trichocarpa*, using combinations of 14 morphological/physiological traits to identify candidate genes involved in these traits (Chhetri et al., 2019).

There have thus been several efforts to integrate various data layers, sometimes for the goal of prioritizing candidate genes, and others for providing biological context for the interpretation of GWAS results.

### Signal Processing

#### Data Representation

In the previous section, we discussed the representation of biological data at network structures, which focuses on relationships between pairs of objects. Here, we discuss the representation of biological data as “signals” and subsequent analysis techniques.

A biological signal represents the response of a variable over some range of input values, which usually have some longitudinal feature, such as a response over increasing time, or a response over increasing distance. Classic examples of biological signals are feature density signals across chromosomes, such as SNP density, gene density, recombination density, and GC content, to name a few (Spencer et al., 2006; Paape et al., 2012; McCormick et al., 2018).

These signals have variation at different scales (i.e., are composed of multiple signals of different frequencies), and signal processing techniques can be used to extract frequency information. McCormick et al. (2018) who used the Fourier Transform to identify a prominent periodicity in SNP density, finding that SNP density peaked with a period of 3 base pairs downstream of coding sequence start sites, which was explained by the positions in the third “wobble” base being under lower selective pressure.

The Fourier transform represents a signal as a linear combination of sine and cosine waves. These are infinite waves and thus the Fourier transform provides no information as to which frequencies are observed at different locations in the signal. The wavelet transform is a newer signal processing technique that addresses this limitation (Leavey et al., 2003).

#### Continuous Wavelet Transform

**Figure 3:**
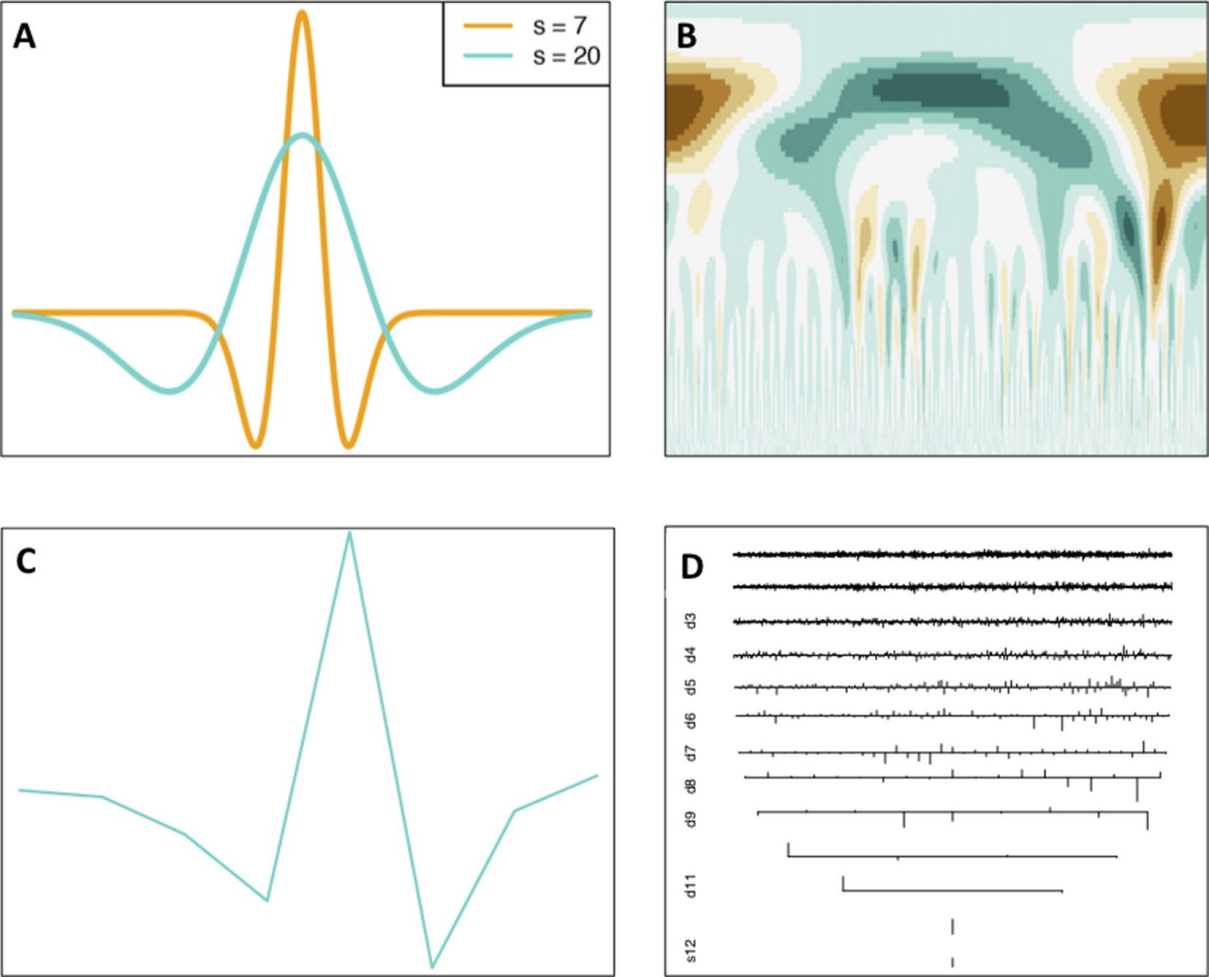
Continuous and discrete wavelet transforms. **(A)** Continuous Ricker Wavelet, **(B)**, CWT coefficient matrix heatmap, **(C)** discrete s8 wavelet, **(D)** DWT coefficients. Wavelet transform images generated using the Wavelet Methods for Time Series Analysis (WMTSA) R package (Percival and Walden, 2000).

The Continuous Wavelet Transform (CWT) is a signal processing technique that expresses a signal as a linear combination of special functions called wavelets. These functions are scaled translations of a mother wavelet function, i.e., different widths and different *x*-axis locations of a particular function. A wavelet *w* is required to have oscillations and is required to “die out,” i.e., the function $\lim_{x\to \infty }w(x)=0$. An example of a wavelet function called the Ricker Wavelet can be seen in **Figure 3A**.

What results from a wavelet transform is a wavelet coefficient *W*(*s*, τ) (Equation 2), for every scale *s* and translation (shift along the *x*-axis) τ (Leavey et al., 2003).

$$W(s,\tau )=\frac{1}{\sqrt{s}}\int f(t)\psi \text{*}\left(\frac{t-\tau }{s}\right)dts$$

This essentially can be interpreted as “sliding” the wavelet of a certain width over the signal, and at each position calculating the integral of the product of the wavelet and the signal over the entire *x*-axis, producing a vector of coefficients. This process is then repeated for multiple widths of the mother wavelet. An example of the CWT applied to the SNP density of *P. trichocarpa* chromosome 1 can be seen in **Figure 3B**. Other visual examples of various mother wavelets and CWT coefficient outputs can be seen in references (Leavey et al., 2003; Mi et al., 2005;Spencer et al., 2006;Dong et al., 2008).

#### Discrete Wavelet Transform

The Discrete Wavelet Transform (DWT) is a sampled version of the CWT and involves sampling of the *x* dimension of the signal and scale dimension of the wavelet (Leavey et al., 2003). This is a dyadic sampling, which results in low-frequency, large scales being sampled sparsely and high-frequency, small scales being sampled densely (Alsberg et al., 1997). The DWT uses discrete wavelet functions (for example, see **Figure 3C**) and produces a series of sets of coefficients with one set of coefficients for each scale computed (**Figure 3D**). DWT coefficients for Palmer Drought Severity Index data across time can be seen in the tutorial by Dong et al. (2008).

#### Wavelet-Based Analysis and Integration of Biological Data

A useful overview of the wavelet transform and previous biological applications prior to 2003, including sequence analysis, protein structure investigation, and expression data analysis to identify periodicities, can be found in the review by Liò (2003). More recent applications of the wavelet transform in biological data analysis are discussed below.

Thurman et al. (2007) performed an investigation to detect “functional domains” of a scale larger than that of within a gene, in the human genome. The wavelet transform was used to smooth density signals of various ENCODE data over various scales. This included transcriptional data, histone acetylation, histone methylation, and DNA replication time. A hidden Markov model was then used to segment the genome into one of two states, namely, state 0 (“repressed”) and state 1 (“active”), particularly signal (Thurman et al., 2007). This was performed separately for each data type and also in a combined fashion. Domains with the state 1 (“active”) classification were enriched in characteristics of “active” chromatin, for example, transcriptional stop/start sites, mRNAs, and CpG islands, among others. However, domains with the state 0 (“repressed”) classification were significantly enriched in signal transduction genes as determined using GO enrichment. TEs in general were evenly distributed across active and repressed domains; however, certain classes of repeats, such as L1 LINE repeats and LTR elements, were enriched in state 0 domains (“repressed” domains) (Thurman et al., 2007).

Shim and Stephens (2015) determined variants that are associated with open chromatin using DNase-seq data from 70 genotyped individuals. Chromatin accessibility vectors are transformed using the DWT prior to associating them to phenotypes. The advantage of this method is that it takes into account the read profile, without having to resort to “artificial” boundaries such as known exon boundaries or sliding windows of a set size.

Machado et al. (2011) performed wavelet analysis of sequence data by transforming DNA sequence into a vector of numbers, with each base pair mapped to a point on one of the axes of the complex plane. The wavelet transform is applied to these sequence vectors and various wavelets are tested. However, no functional interpretations of results were discussed.

Biological signals can have different relationships with each other depending on the scale at which one is looking. While two features may be correlated at certain scales, they may be anti-correlated at others. Keitt and Urban (2005) introduced *wavelet-coefficient regression*, in which wavelet transforms are applied to dependent and independent variables before performing regression analysis, allowing for scale-specific inference. Spencer et al. (2006) used this kind of approach, applying the DWT and linear model analysis to investigate scale-specific relationships between various genomic features including genomic signals of recombination, divergence, diversity, GC content, and gene content in 1-kb regions across human chromosome 20. The DWT was performed on each of these signals, and the correlation between the wavelet coefficients of features at each scale was calculated to identify scale-specific correlations (Spencer et al., 2006). Paape et al. (2012) applied the same approach as Spencer et al. (2006), using the wavelet transform followed by linear model analysis to identify genomic features that correlate with recombination in *M. truncatula*. The wavelet correlation results revealed a negative correlation between recombination and the distance to the centromere, which had not been found in several other organisms (Paape et al., 2012). Very recently, Fernández et al. (2018) applied the wavelet transform in an application for visualizing DNA methylation data at various scales/resolutions.

Representation of multiple *P. trichocarpa* data layers as signals was performed by Vining et al. (2012), in which methylation density signals of various *P. trichocarpa* tissues were overlapped. In addition, methylation density signals were overlaid with gene density signals and k-mer density signals, and approximate centromere locations on a subset of the *P. trichocarpa* chromosomes were visually reported (Vining et al., 2012).

Weighill et al. (2019b) performed the first application of the wavelet transform to multi-omics data layering in *Poplar*. Weighill et al. (2019b) made use of the methylation data from Vining et al. (2012) and Vining et al. (2013), as well as variant data and genome annotations to construct gene density, variant density, and methylation density profiles for *P. trichocarpa* (Weighill et al., 2019b). The wavelet transform was used to characterize the variation in these signals at multiple scales, extract the relevant centromere/pericentromere scales of variation, and predict the locations of the centromere on all 19 *P. trichocarpa* chromosomes, making use of information from variant density and methylation density signals.

## Concluding Remarks and Future Prospects

In this review, we have discussed large-scale ‘omics data types, multi-omics studies, as well as network-based analysis/integration techniques and wavelet-based multi-scale analysis and comparisons, all with a particular focus on investigations performed in *Populus*. **Table 3** summarizes examples of multi-omic/data integration studies in *Populus*. While many such studies have been performed over the last decade, few studies involve the integration of multiple data types in a combined analysis, as opposed to a sequential analysis.

**Table 3:** Examples of multi-omic/data integration studies in *Populus* species.

Species	Data Types/Layers	Reference
*P. trichocarpa*	Transcriptomics, metabolomics, biomass/sugar release	Zhang et al. (2019)
*P. trichocarpa*	Genomic, transcriptomic, proteomic, fluxomic, wood chemical property phenotypes	Wang et al. (2018b)
*P. tremula × P. tremuloides*	Transcriptome, proteome, GC-MS metabolome, LC-MS metabolome, pyrolysis-GC MS metabolome	Obudulu et al. (2018)
*P. trichocarpa*	Transcriptomics, co-expression, genotype, callus phenotype (GWAS)	Tuskan et al. (2018)
*P. trichocarpa*	Metabolomics, genotype, transcriptomics, GWAS, eQTL, co-expression	Zhang et al. (2018)
*P. deltoides*	Metabolomics, microbiome	Veach et al. (2018)
*P. trichocarpa*	Co-expression, protein–protein interaction, population genotype	Tian et al. (2017)
*P. trichocarpa*	Methylation, transcript expression, miRNAs	Schönberger et al. (2016)
*P. tremuloides and Laccaria*	Transcriptomics, protein–protein interactions,	Larsen et al. (2016)
*P. balsamifera*	Transcriptomics, metabolomics	Hamanishi et al. (2015)
*P. trichocarpa and P. deltoides*	Metabolomics, transcriptomics	Tschaplinski et al. (2014)
*P. trichocarpa*	Genotype, phenotype (GWAS)	McKown et al. (2014)
*P. trichocarpa*	Methylome (bisulfite sequencing), transcriptomics	Liang et al. (2014)
*P. trichocarpa*	Genotype, phenotype (GWAS)	Evans et al. (2014)
*P. trichocarpa*	Methylome (MeDIP-seq), transcriptomics	Vining et al. (2013)
*P. trichocarpa*	Open chromatin, methylome	Lafon-Placette et al. (2013)
*P. trichocarpa*	Methylome (MeDIP-seq), transcriptomics, transposable elements	Vining et al. (2012)
*P. trichocarpa*	Genotype, repeat elements, methylation, recombination	Slavov et al. (2012)
*Populus euphratica and Populus × canescens*	Transcriptomics, metabolomics	Janz et al. (2010)
*P. tremula × P. tremuloides*	Transcriptomics, metabolomics, proteomics	Bylesjo et al. (2008)
*P. tremula × P. tremuloides*	Transcriptomics, metabolomics	Bylesjö et al. (2007)
*P. deltoides × P. nigra and P. deltoides × P. trichocarpa*	Genotypes, metabolites (mQTLs)	Morreel et al. (2006)

A vast collection of different data types has been generated for *P. trichocarpa*. As described in this review, the genome has been sequenced and annotated (Tuskan et al., 2006), and the assembly is currently in its fourth version of revision. Approximately ∼1,300 *P. trichocarpa* genotypes have been propagated in four different common gardens (Tuskan et al., 2011; Slavov et al., 2012; Evans et al., 2014) and have been resequenced. This has provided a large set of ∼28,000,000 single-nucleotide polymorphisms (SNPs) that have recently been publicly released (DOI 10.13139/OLCF/1411410). Many molecular phenotypes measured through untargeted metabolomics, RNA-Seq, ionomics, and pyMBMS, as well as physical properties (Porth et al., 2013) measured in this population have provided an unparalleled resource for GWASs [for example, see McKown et al. (2014)]. DNA methylation data in the form of MeDIP (Methyl-DNA immunoprecipitation)-seq have been performed on 10 different *P. trichocarpa* tissues (Vining et al., 2012).

The availability of public data as well as access to high-performance computing resources provides an opportunity for the large-scale, concurrent analysis of these multiple datasets in order to profile and characterize the *P. trichocarpa* genome; identify complex gene–phenotype relationships, such as pleiotropy and epistasis, from genome-wide association data; as well as perform large-scale target gene identification from integrated multi-omics datasets. Multi-scale analysis could allow for the interrogation of scale-specific relationships between various genomic features and could potentially provide insights into the evolutionary history of the *P. trichocarpa* genome. Integrated analysis of various ‘omics data layers will expand the system-wide knowledge of *Populus* species, which is necessary for the continued development of *Populus* as a model tree species and as a domesticated, efficient biofuel feedstock.

## Author Contributions

DW and DJ planned the manuscript. DW performed the research and wrote the manuscript. DJ, TT, and GT provided editorial feedback.

## Funding

Funding was provided by the Center for Bioenergy Innovation (CBI). The Center for Bioenergy Innovation is a U.S. Department of Energy Bioenergy Research Center supported by the Office of Biological and Environmental Research in the DOE Office of Science.

This research was also supported by the Plant–Microbe Interfaces Scientific Focus Area (http://pmi.ornl.gov) in the Genomic Science Program, the Office of Biological and Environmental Research (BER) in the U.S. Department of Energy Office of Science.

An award of computer time was provided by the INCITE program. This research used resources of the Oak Ridge Leadership Computing Facility (OLCF) at the Oak Ridge National Laboratory, which is supported by the Office of Science of the U.S. Department of Energy under Contract No. DE-AC05-00OR22725.

## Conflict of Interest Statement

The authors declare that the research was conducted in the absence of any commercial or financial relationships that could be construed as a potential conflict of interest.

## References

[B1] Abel H. J. Duncavage E. J. (2013). Detection of structural DNA variation from next generation sequencing data: a review of informatic approaches. Cancer Genet. 206, 432–440. 10.1016/j.cancergen.2013.11.002 24405614 PMC4441822

[B2] Abraham P. E. Garcia B. J. Gunter L. E. Jawdy S. S. Engle N. Yang X. (2018). Quantitative proteome profile of water deficit stress responses in eastern cottonwood (*Populus deltoides*) leaves. PloS One 13, e0190019. 10.1371/journal.pone.0190019 29447168 PMC5813909

[B3] Akula N. Baranova A. Seto D. Solka J. Nalls M. A. Singleton A. (2011). A network-based approach to prioritize results from genome-wide association studies. PloS One 6, e24220. 10.1371/journal.pone.0024220 21915301 PMC3168369

[B4] Akulenko R. Helms V. (2013). DNA co-methylation analysis suggests novel functional associations between gene pairs in breast cancer samples. Hum. Mol. Genet. 22, 3016–3022. 10.1093/hmg/ddt158 23571108

[B5] Alsberg B. K. Woodward A. M. Kell D. B. (1997). An introduction to wavelet transforms for chemometricians: a time–frequency approach. Chemom. Intell. Lab. Syst. 37, 215–239. 10.1016/S0169-7439(97)00029-4

[B6] Aoki K. Ogata Y. Shibata D. (2007). Approaches for extracting practical information from gene co-expression networks in plant biology. Plant Cell Physiol. 48, 381–390. 10.1093/pcp/pcm013 17251202

[B7] Arneson D. Bhattacharya A. Shu L. Mäkinen V.-P. Yang X. (2016). Mergeomics: a web server for identifying pathological pathways, networks, and key regulators *via* multidimensional data integration. BMC Genomics 17, 722. 10.1186/s12864-016-3057-8 27612452 PMC5016927

[B8] Astle W. Balding D. J., (2009). Population structure and cryptic relatedness in genetic association studies. Stat. Sci. 24, 451–471. 10.1214/09-STS307

[B9] Bajic M. Maher K. A. Deal R. B. (2018) “Identification of Open Chromatin Regions in Plant Genomes Using ATAC-Seq”. In Plant Chromatin Dynamics. Methods in Molecular Biology. 1675 New York, NY, Humana Press. 10.1007/978-1-4939-7318-7_12 PMC5693289 29052193

[B10] Bao H. Li E. Mansfield S. D. Cronk Q. C. El-Kassaby Y. A. Douglas C. J. (2013). The developing xylem transcriptome and genome-wide analysis of alternative splicing in *Populus trichocarpa* (black cottonwood) populations. BMC Genomics 14, 359. 10.1186/1471-2164-14-359 23718132 PMC3680236

[B11] Barabasi A.-L. Oltvai Z. N. (2004). Network biology: understanding the cell’s functional organization. Nat. Rev. Genet. 5, 101–113. 10.1038/nrg1272 14735121

[B12] Bartlett A. O’Malley R. C. Huang S.-s. C. Galli M. Nery J. R. Gallavotti A. (2017). Mapping genome-wide transcription-factor binding sites using DAP-seq. Nat. Protoc. 12, 1659. 10.1038/nprot.2017.055 28726847 PMC5576341

[B13] Bartlett T. E. Olhede S. C. Zaikin A. (2014). A DNA methylation network interaction measure, and detection of network oncomarkers. PloS One 9, e84573. 10.1371/journal.pone.0084573 24400102 PMC3882261

[B14] Benedito V. A. Torres-Jerez I. Murray J. D. Andriankaja A. Allen S. Kakar K. (2008). A gene expression atlas of the model legume *Medicago truncatula*. Plant J. 55, 504–513. 10.1111/j.1365-313X.2008.03519.x 18410479

[B15] Bennetzen J. L. Wang H. (2014). The contributions of transposable elements to the structure, function, and evolution of plant genomes. Annu. Rev. Plant Biol. 65, 505–530. 10.1146/annurev-arplant-050213-035811 24579996

[B16] Bock C. (2012). Analysing and interpreting DNA methylation data. Nat. Rev. Genet. 13, 705. 10.1038/nrg3273 22986265

[B17] Bryan A. C. Zhang J. Guo J. Ranjan P. Singan V. Barry K. (2018). A variable polyglutamine repeat affects subcellular localization and regulatory activity of a *Populus* ANGUSTIFOLIA protein. G3: Genes, Genomes, Genet. 8, 2631–2641. 10.1534/g3.118.200188 PMC6071607 29884614

[B18] Buenrostro J. D. Giresi P. G. Zaba L. C. Chang H. Y. Greenleaf W. J. (2013). Transposition of native chromatin for fast and sensitive epigenomic profiling of open chromatin, DNA-binding proteins and nucleosome position. Nat. Methods 10, 1213. 10.1038/nmeth.2688 24097267 PMC3959825

[B19] Buenrostro J. D. Wu B. Chang H. Y. Greenleaf W. J. (2015). ATAC-seq: amethod for assaying chromatin accessibility genome-wide. Curr. Protoc. Mol. Biol. 109, 21–29. 10.1002/0471142727.mb2129s109 PMC4374986 25559105

[B20] Bunyavanich S. Schadt E. E. Himes B. E. Lasky-Su J. Qiu W. Lazarus R. (2014). Integrated genome-wide association, coexpression network, and expression single nucleotide polymorphism analysis identifies novel pathway in allergic rhinitis. BMC Med. Genomics 7, 48. 10.1186/1755-8794-7-48 25085501 PMC4127082

[B21] Bush W. S. Moore J. H. (2012). Genome-wide association studies. PLoS Comput. Biol. 8, e1002822. 10.1371/journal.pcbi.1002822 23300413 PMC3531285

[B22] Bylesjö M. Eriksson D. Kusano M. Moritz T. Trygg J. (2007). Data integration in plant biology: the O2PLS method for combined modeling of transcript and metabolite data. Plant J. 52, 1181–1191. 10.1111/j.1365-313X.2007.03293.x 17931352

[B23] Bylesjo M. Nilsson R. Srivastava V. Gronlund A. Johansson A. I. Jansson S. (2008). Integrated analysis of transcript, protein and metabolite data to study lignin biosynthesis in hybrid aspen. J. Proteome Res. 8, 199–210. 10.1021/pr800298s 19053836

[B24] Calabrese G. M. Mesner L. D. Stains J. P. Tommasini S. M. Horowitz M. C. Rosen C. J. (2017). Integrating GWAS and co-expression network data identifies bone mineral density genes SPTBN1 and MARK3 and an osteoblast functional module. Cell Syst. 4, 46–59. 10.1016/j.cels.2016.10.014 27866947 PMC5269473

[B25] Capy P. Gasperi G. Biémont C. Bazin C. (2000). Stress and transposable elements: co-evolution or useful parasites? Heredity 85, 101. 10.1046/j.1365-2540.2000.00751.x 11012710

[B26] Chao Q. Gao Z.-F. Zhang D. Zhao B.-G. Dong F.-Q. Fu C.-X. (2019). The developmental dynamics of the *Populus* stem transcriptome. Plant Biotechnol. J. 17, 206–219. 10.1111/pbi.12958 29851301 PMC6330540

[B27] Chen S. Harmon A. C. (2006). Advances in plant proteomics. Proteomics 6, 5504–5516. 10.1002/pmic.200600143 16972296

[B28] Chhetri H. B. Macaya-Sanz D. Kainer D. Biswal A. K. Evans L. M. Chen J.-G. (2019). Multitrait genome-wide association analysis of *Populus trichocarpa* identifies key polymorphisms controlling morphological and physiological traits. New Phytol. 223, 293–309. 10.1111/nph.15777 30843213

[B29] Chinnusamy V. Zhu J.-K. (2009). Epigenetic regulation of stress responses in plants. Curr. Opin. Plant Biol. 12, 133–139. 10.1016/j.pbi.2008.12.006 19179104 PMC3139470

[B30] Climer S. Templeton A. R. Zhang W. (2014a). Allele-specific network reveals combinatorial interaction that transcends small effects in psoriasis GWAS. PLOS Comput. Biol. 10, e1003766. 10.1371/journal.pcbi.1003766 25233071 PMC4168982

[B31] Climer S. Templeton A. R. Zhang W. (2015). Human gephyrin is encompassed within giant functional noncoding yin–yang sequences. Nat. Commun. 6, 6534. 10.1038/ncomms7534 25813846 PMC4380243

[B32] Climer S. Yang W. Fuentes L. Dávila-Román V. G. Gu C. C. (2014b). A Custom Correlation Coefficient (CCC) approach for fast identification of multi-SNP association patterns in genome-wide SNPs data. Genet. Epidemiol. 38, 610–621. 10.1002/gepi.21833 25168954 PMC4190009

[B33] Cossu R. M. Buti M. Giordani T. Natali L. Cavallini A. (2012). A computational study of the dynamics of LTR retrotransposons in the *Populus trichocarpa* genome. Tree Genet. Genomes 8, 61–75. 10.1007/s11295-011-0421-3

[B34] Darabos C. Harmon S. H. Moore J. H. (2014). Using the bipartite human phenotype network to reveal pleiotropy and epistasis beyond the gene. Pac. Symp. Biocomput. (World Sci.) 19, 188–199. 10.1142/9789814583220_0019 PMC3900286 24297546

[B35] Dash M. Yordanov Y. S. Georgieva T. Wei H. Busov V. (2018). Gene network analysis of poplar root transcriptome in response to drought stress identifies a PtaJAZ3PtaRAP2. 6-centered hierarchical network. PloS One 13, e0208560. 10.1371/journal.pone.0208560 30540849 PMC6291141

[B36] Dimitrakopoulos C. Hindupur S. K. Häfliger L. Behr J. Montazeri H. Hall M. N. (2018). Network-based integration of multi-omics data for prioritizing cancer genes. Bioinformatics 34, 2441–2448. 10.1093/bioinformatics/bty148 29547932 PMC6041755

[B37] Dong X. Nyren P. Patton B. Nyren A. Richardson J. Maresca T. (2008). Wavelets for agriculture and biology: a tutorial with applications and outlook. BioScience 58, 445–453. 10.1641/B580512

[B38] Druka A. Muehlbauer G. Druka I. Caldo R. Baumann U. Rostoks N. (2006). An atlas of gene expression from seed to seed through barley development. Funct. Integr. Genomics 6, 202–211. 10.1007/s10142-006-0025-4 16547597

[B39] Durand T. C. Sergeant K. Renaut J. Planchon S. Hoffmann L. Carpin S. (2011). Poplar under drought: comparison of leaf and cambial proteomic responses. J. Proteomics 74, 1396–1410. 10.1016/j.jprot.2011.03.013 21439416

[B40] Emamjomeh A. Robat E. S. Zahiri J. Solouki M. Khosravi P. (2017). Gene co-expression network reconstruction: a review on computational methods for inferring functional information from plant-based expression data. Plant Biotechnol. Rep. 11, 71–86. 10.1007/s11816-017-0433-z

[B41] Enright A. J. Van Dongen S. Ouzounis C. A. (2002). An efficient algorithm for large-scale detection of protein families. Nucleic Acids Res. 30, 1575–1584. 10.1093/nar/30.7.1575 11917018 PMC101833

[B42] Evans L. M. Slavov G. T. Rodgers-Melnick E. Martin J. Ranjan P. Muchero W. (2014). Population genomics of *Populus trichocarpa* identifies signatures of selection and adaptive trait associations. Nat. Genet. 46, 1089–1096. 10.1038/ng.3075 25151358

[B43] Fadista J. Manning A. K. Florez J. C. Groop L. (2016). The (in) famous GWAS P-value threshold revisited and updated for low-frequency variants. Eur. J. Hum. Genet. 24, 1202. 10.1038/ejhg.2015.269 26733288 PMC4970684

[B44] Fagny M. Paulson J. N. Kuijjer M. L. Sonawane A. R. Chen C.-Y. Lopes-Ramos C. M. (2017). Exploring regulation in tissues with eQTL networks. Proc. Nat. Acad. Sci. 114, E7841–E7850. 10.1073/pnas.1707375114 28851834 PMC5604022

[B45] Farber C. R. (2013). Systems-level analysis of genome-wide association data. G3: Genes, Genomes, Genet. 3, 119–129. 10.1534/g3.112.004788 PMC3538337 23316444

[B46] Fasoli M. Dal Santo S. Zenoni S. Tornielli G. Farina L. Zamboni A. Pezzotti M. (2012). The Grapevine Expression Atlas Reveals a Deep Transcriptome Shift Driving the Entire Plant into a Maturation Program. The Plant Cell. 24(9), 3489-3505. 10.1105/tpc.112.100230 22948079 PMC3480284

[B47] Fernández L. Pérez M. Orduña J. M. (2018). Visualization of DNA methylation results through a GPU-based parallelization of the wavelet transform. J. Supercomput. 75 (3), 1496–1509. 10.1007/s11227-018-2670-5

[B48] Finnegan E. Genger R. Peacock W. Dennis E. (1998). DNA methylation in plants. Annu. Rev. Plant Biol. 49, 223–247. 10.1146/annurev.arplant.49.1.223 15012234

[B49] Flint-Garcia S. A. Thornsberry J. M. IV B. (2003). Structure of linkage disequilibrium in plants. Annu. Rev. Plant Biol. 54, 357–374 10.1146/annurev.arplant.54.031902.134907 14502995

[B50] Frommer M. McDonald L. E. Millar D. S. Collis C. M. Watt F. Grigg G. W. (1992). A genomic sequencing protocol that yields a positive display of 5-methylcytosine residues in individual DNA strands. Proc. Nat. Acad. Sci. 89, 1827–1831. 10.1073/pnas.89.5.1827 1542678 PMC48546

[B51] Fukushima A. Kusano M. (2014). A network perspective on nitrogen metabolism from model to crop plants using integrated ‘omics’ approaches. J. Exp. Bot. 65, 5619–5630. 10.1093/jxb/eru322 25129130

[B52] Gaut B. S. Wright S. I. Rizzon C. Dvorak J. Anderson L. K. (2007). Recombination: an underappreciated factor in the evolution of plant genomes. Nat. Rev. Genet. 8, 77. 10.1038/nrg1970 17173059

[B53] Gene Ontology Consortium (2004). The Gene Ontology (GO) database and informatics resource. Nucleic Acids Res. 32, D258–D261. 10.1093/nar/gkh036 14681407 PMC308770

[B54] Geraldes A. Difazio S. Slavov G. Ranjan P. Muchero W. Hannemann J. (2013). A 34K SNP genotyping array for *Populus trichocarpa*: design, application to the study of natural populations and transferability to other *Populus* species. Mol. Ecol. Resour. 13, 306–323. 10.1111/1755-0998.12056 23311503

[B55] Gligorijević V. Pržulj N. (2015). Methods for biological data integration: perspectives and challenges. J. Royal Soc. Interface 12, 20150571. 10.1098/rsif.2015.0571 PMC4685837 26490630

[B56] Goh K.-I. Cusick M. E. Valle D. Childs B. Vidal M. Barabási A.-L. (2007). The human disease network. Proc. Nat. Acad. Sci. 104, 8685–8690. 10.1073/pnas.0701361104 17502601 PMC1885563

[B57] Golumbic M. (2004).“Annals of discrete mathematics” in Algorithmic graph theory and perfect graphs. 2nd ed Amsterdam; Boston: Elsevier. 10.1016/S0167-5060(04)80059-1

[B58] Goodstein D. M. Shu S. Howson R. Neupane R. Hayes R. D. Fazo J. (2012). Phytozome: a comparative platform for green plant genomics. Nucleic Acids Res. 40, D1178–D1186. 10.1093/nar/gkr944 22110026 PMC3245001

[B59] Grönlund A. Bhalerao R. P. Karlsson J. (2009). Modular gene expression in Poplar: a multilayer network approach. New Phytol. 181, 315–322. 10.1111/j.1469-8137.2008.02668.x 19121030

[B60] Gutiérrez R. A. Lejay L. V. Dean A. Chiaromonte F. Shasha D. E. Coruzzi G. M. (2007). Qualitative network models and genome-wide expression data define carbon/nitrogen-responsive molecular machines in *Arabidopsis*. Genome Biol. 8, R7. 10.1186/gb-2007-8-1-r7 17217541 PMC1839130

[B61] Ha M. J. Baladandayuthapani V. Do K.-A. (2015). DINGO: differential network analysis in genomics. Bioinformatics 31, 3413–3420. 10.1093/bioinformatics/btv406 26148744 PMC4751246

[B62] Hamanishi E. T. Barchet G. L. Dauwe R. Mansfield S. D. Campbell M. M. (2015). Poplar trees reconfigure the transcriptome and metabolome in response to drought in a genotype-and time-of-day-dependent manner. BMC Genomics 16, 329. 10.1186/s12864-015-1535-z 25895923 PMC4437445

[B63] Hassani-Pak K. (2017) KnetMiner — An integrated data platform for gene mining and biological knowledge discovery. Bielefeld: Universität Bielefeld.

[B64] Hassani-Pak K. Castellote M. Esch M. Hindle M. Lysenko A. Taubert J. (2016). Developing integrated crop knowledge networks to advance candidate gene discovery. Appl. Transl. Genomics 11, 18–26. 10.1016/j.atg.2016.10.003 PMC5167366 28018846

[B65] Hassani-Pak K. Rawlings C. (2017). Knowledge discovery in biological databases for revealing candidate genes linked to complex phenotypes. J. Integr. Bioinf. 14 (1), 803–809. 10.1515/jib-2016-0002 PMC6042805 28609292

[B66] Horvath S. Dong J. (2008). Geometric interpretation of gene coexpression network analysis. PLoS Comput. Biol. 4, e1000117. 10.1371/journal.pcbi.1000117 18704157 PMC2446438

[B67] Hu H. Scheben A. Edwards D. (2018). Advances in integrating genomics and bioinformatics in the plant breeding pipeline. Agriculture 8, 75. 10.3390/agriculture8060075

[B68] Ingvarsson P. K. Hvidsten T. R. Street N. R. (2016). Towards integration of population and comparative genomics in forest trees. New Phytol. 212, 338–344. 10.1111/nph.14153 27575589

[B69] Jacinto F. V. Ballestar E. Esteller M. (2008). Methyl-DNA immunoprecipitation (MeDIP): hunting down the DNA methylome. Biotechniques 44, 35–43. 10.2144/000112708 18254377

[B70] Jansson S. Douglas C. J. (2007). *Populus*: a model system for plant biology. Annu. Rev. Plant Biol. 58, 435–458. 10.1146/annurev.arplant.58.032806.103956 17280524

[B71] Janz D. Behnke K. Schnitzler J.-P. Kanawati B. Schmitt-Kopplin P. Polle A. (2010). Pathway analysis of the transcriptome and metabolome of salt sensitive and tolerant poplar species reveals evolutionary adaption of stress tolerance mechanisms. BMC Plant Biol. 10, 150. 10.1186/1471-2229-10-150 20637123 PMC3095294

[B72] Johnson R. C. Nelson G. W. Troyer J. L. Lautenberger J. A. Kessing B. D. Winkler C. A. (2010). Accounting for multiple comparisons in a genome-wide association study (GWAS). BMC Genomics 11, 724. 10.1186/1471-2164-11-724 21176216 PMC3023815

[B73] Joubert W. Nance J. Climer S. Weighill D. Jacobson D. (2019). Parallel accelerated custom correlation coefficient calculations for genomics applications. Parallel Comput. 84, 15–23. 10.1016/j.parco.2019.02.003

[B74] Joubert W. Weighill D. Kainer D. Climer S. Justice A. Fagnan K. (2018). Attacking the opioid epidemic: determining the epistatic and pleiotropic genetic architectures for chronic pain and opioid addiction. SC '18 Proceedings of the International Conference for High Performance Computing, Networking, Storage, and Analysis; 2018 November 11–16; NJ, USA: IEEE Press Piscataway, 57. 10.1109/SC.2018.00060

[B75] Kaling M. Kanawati B. Ghirardo A. Albert A. Winkler J. B. Heller W. (2015). UV-B mediated metabolic rearrangements in poplar revealed by non-targeted metabolomics. Plant. Cell Environ. 38, 892–904. 10.1111/pce.12348 24738572

[B76] Keitt T. H. Urban D. L. (2005). Scale-specific inference using wavelets. Ecology 86, 2497–2504. 10.1890/04-1016

[B77] Kejnovsky E. Hawkins J. S. Feschotte C. (2012). “Plant transposable elements: biology and evolution,” in Plant Genome Diversity. 1, 17–34. Vienna: Springer. 10.1007/978-3-7091-1130-7_2

[B78] Klein S. J. O’Neill R. J. (2018). Transposable elements: genome innovation, chromosome diversity, and centromere conflict. Chromosome Res., 26(1-2), 5–23. 10.1007/s10577-017-9569-5 29332159 PMC5857280

[B79] Kogelman L. J. Kadarmideen H. N. (2014). Weighted Interaction SNP Hub (WISH) network method for building genetic networks for complex diseases and traits using whole genome genotype data. BMC Syst. Biol. 8, S5. 10.1186/1752-0509-8-S2-S5 PMC4101698 25032480

[B80] Korte A. Farlow A. (2013). The advantages and limitations of trait analysis with GWAS: a review. Plant Methods 9, 29. 10.1186/1746-4811-9-29 23876160 PMC3750305

[B81] Krishnan A. Taroni J. N. Greene C. S. (2016). Integrative networks illuminate biological factors underlying gene–disease associations. Curr. Genet. Med. Rep. 4, 155–162. 10.1007/s40142-016-0102-5

[B82] Krueger F. Kreck B. Franke A. Andrews S. R. (2012). DNA methylome analysis using short bisulfite sequencing data. Nat. Methods 9, 145. 10.1038/nmeth.1828 22290186

[B83] Lafon-Placette C. Faivre-Rampant P. Delaunay A. Street N. Brignolas F. Maury S. (2013). Methylome of DNase I sensitive chromatin in *Populus trichocarpa* shoot apical meristematic cells: a simplified approach revealing characteristics of gene-body DNA methylation in open chromatin state. New Phytol. 197, 416–430. 10.1111/nph.12026 23253333

[B84] LaFramboise T. (2009). Single nucleotide polymorphism arrays: a decade of biological, computational and technological advances. Nucleic Acids Res. 37, 4181–4193. 10.1093/nar/gkp552 19570852 PMC2715261

[B85] Laird P. W. (2010). Principles and challenges of genome-wide DNA methylation analysis. Nat. Rev. Genet. 11, 191. 10.1038/nrg2732 20125086

[B86] Lämke J. Bäurle I. (2017). Epigenetic and chromatin-based mechanisms in environmental stress adaptation and stress memory in plants. Genome Biol. 18, 124. 10.1186/s13059-017-1263-6 28655328 PMC5488299

[B87] Langfelder P. Horvath S. (2008). WGCNA: an R package for weighted correlation network analysis. BMC Bioinf. 9, 559. 10.1186/1471-2105-9-559 PMC2631488 19114008

[B88] Larsen P. E. Sreedasyam A. Trivedi G. Desai S. Dai Y. Cseke L. J. (2016). Multi-omics approach identifies molecular mechanisms of plant–fungus mycorrhizal interaction. Front. Plant Sci. 6, 1061. 10.3389/fpls.2015.01061 26834754 PMC4717292

[B89] Law J. A. Jacobsen S. E. (2010). Establishing, maintaining and modifying DNA methylation patterns in plants and animals. Nat. Rev. Genet. 11, 204. 10.1038/nrg2719 20142834 PMC3034103

[B90] Leavey C. James M. Summerscales J. Sutton R. (2003). An introduction to wavelet transforms: a tutorial approach. Insight-Non-Destr. Test. Condition Monit. 45, 344–353. 10.1784/insi.45.5.344.52875

[B91] Lee T. Kim H. Lee I. (2015). Network-assisted crop systems genetics: network inference and integrative analysis. Curr. Opin. Plant Biol. 24, 61–70. 10.1016/j.pbi.2015.02.001 25698380

[B92] Leiserson M. D. Eldridge J. V. Ramachandran S. Raphael B. J. (2013). Network analysis of GWAS data. Curr. Opin. Genet. Dev. 23, 602–610. 10.1016/j.gde.2013.09.003 24287332 PMC3867794

[B93] Levine M.E. Langfelder P. Horvath S. (2017) A Weighted SNP Correlation Network Method for Estimating Polygenic Risk Scores. In: Tatarinova T. Nikolsky Y. (eds) Biological Networks and Pathway Analysis. Methods in Molecular Biology, vol 1613 New York, NY, Humana Press. 10.1007/978-1-4939-7027-8_10 PMC5998804 28849564

[B94] Li Y. Pearl S. A. Jackson S. A. (2015). Gene networks in plant biology: approaches in reconstruction and analysis. Trends Plant Sci. 20, 664–675. 10.1016/j.tplants.2015.06.013 26440435

[B95] Liang D. Zhang Z. Wu H. Huang C. Shuai P. Ye C.-Y. (2014). Single-base-resolution methylomes of *Populus trichocarpa* reveal the association between DNA methylation and drought stress. BMC Genet. 15, S9. 10.1186/1471-2156-15-S1-S9 PMC4118614 25080211

[B96] Liò P. (2003). Wavelets in bioinformatics and computational biology: State of art and perspectives. Bioinformatics 19, 2–9. 10.1093/bioinformatics/19.1.2 12499286

[B97] Liu C. Lu F. Cui X. Cao X. (2010). Histone methylation in higher plants. Annu. Rev. Plant Biol. 61, 395–420. 10.1146/annurev.arplant.043008.091939 20192747

[B98] Liu J. Hai G. Wang C. Cao S. Xu W. Jia Z. (2015). Comparative proteomic analysis of *Populus trichocarpa* early stem from primary to secondary growth. J. Proteomics 126, 94–108. 10.1016/j.jprot.2015.05.032 26047713

[B99] Liu J. Ye M. Zhu S. Jiang L. Sang M. Gan J. (2018). Two-stage identification of SNP effects on dynamic poplar growth. Plant J. 93, 286–296. 10.1111/tpj.13777 29168265

[B100] Liu Q. Ding C. Chu Y. Chen J. Zhang W. Zhang B. (2016a). PoplarGene: poplar gene network and resource for mining functional information for genes from woody plants. Sci. Rep. 6, 31356. 10.1038/srep31356 27515999 PMC4981870

[B101] Liu Y. Beyer A. Aebersold R. (2016b). On the dependency of cellular protein levels on mRNA abundance. Cell 165, 535–550. 10.1016/j.cell.2016.03.014 27104977

[B102] Lu Z. Hofmeister B. T. Vollmers C. DuBois R. M. Schmitz R. J. (2016). Combining ATAC-seq with nuclei sorting for discovery of cis-regulatory regions in plant genomes. Nucleic Acids Res. 45, e41–e41. 10.1093/nar/gkw1179 PMC5389718 27903897

[B103] Luo J. Xia W. Cao P. Xiao Z. Zhang Y. Liu M. (2019). Integrated transcriptome analysis reveals plant hormones jasmonic acid and salicylic acid coordinate growth and defense responses upon fungal infection in poplar. Biomolecules 9, 12. 10.3390/biom9010012 PMC6358764 30609760

[B104] Lusser A. Kölle D. Loidl P. (2001). Histone acetylation: lessons from the plant kingdom. Trends Plant Sci. 6, 59–65. 10.1016/S1360-1385(00)01839-2 11173289

[B105] Machado J. T. Costa A. C. Quelhas M. D. (2011). Wavelet analysis of human DNA. Genomics 98, 155–163. 10.1016/j.ygeno.2011.05.010 21672622

[B106] Maher K. A. Bajic M. Kajala K. Reynoso M. Pauluzzi G. West D. A. (2018). Profiling of accessible chromatin regions across multiple plant species and cell types reveals common gene regulatory principles and new control modules. Plant Cell 30, 15–36. 10.1105/tpc.17.00581 29229750 PMC5810565

[B107] Mascagni F. Usai G. Natali L. Cavallini A. Giordani T. (2018). A comparison of methods for LTR-retrotransposon insertion time profiling in the *Populus trichocarpa* genome. Caryologia 71, 85–92. 10.1080/00087114.2018.1429749

[B108] McCormick R. F. Truong S. K. Sreedasyam A. Jenkins J. Shu S. Sims D. (2018). The *Sorghum bicolor* reference genome: improved assembly, gene annotations, a transcriptome atlas, and signatures of genome organization. Plant J. 93, 338–354. 10.1111/tpj.13781 29161754

[B109] McKenna A. Hanna M. Banks E. Sivachenko A. Cibulskis K. Kernytsky A. (2010). The Genome Analysis Toolkit: a MapReduce framework for analyzing next-generation DNA sequencing data. Genome Res. 20 (9), 1297–1303. 10.1101/gr.107524.110 20644199 PMC2928508

[B110] McKown A. D. Klápště J. Guy R. D. Geraldes A. Porth I. Hannemann J. (2014). Genome-wide association implicates numerous genes underlying ecological trait variation in natural populations of *Populus trichocarpa*. New Phytol. 203, 535–553. 10.1111/nph.12815 24750093

[B111] Mering C.v. Huynen M. Jaeggi D. Schmidt S. Bork P. Snel B. (2003). STRING: a database of predicted functional associations between proteins. Nucleic Acids Res. 31, 258–261. 10.1093/nar/gkg034 12519996 PMC165481

[B112] Mi X. Ren H. Ouyang Z. Wei W. Ma K. (2005). The use of the Mexican Hat and the Morlet wavelets for detection of ecological patterns. Plant Ecol. 179, 1–19. 10.1007/s11258-004-5089-4

[B113] Mizrachi E. Verbeke L. Christie N. Fierro A. C. Mansfield S. D. Davis M. F. (2017). Network-based integration of systems genetics data reveals pathways associated with lignocellulosic biomass accumulation and processing. Proc. Nat. Acad. Sci. 114, 1195–1200. 10.1073/pnas.1620119114 28096391 PMC5293113

[B114] Morreel K. Goeminne G. Storme V. Sterck L. Ralph J. Coppieters W. (2006). Genetical metabolomics of flavonoid biosynthesis in *Populus*: a case study. Plant J. 47, 224–237. 10.1111/j.1365-313X.2006.02786.x 16774647

[B115] Movahedi S. VanBel M. Heyndrickx K. S. Vandepoele K. (2012). Comparative co-expression analysis in plant biology. Plant Cell Environ. 35, 1787–1798. 10.1111/j.1365-3040.2012.02517.x 22489681

[B116] Natali L. Cossu R. M. Mascagni F. Giordani T. Cavallini A. (2015). A survey of Gypsy and Copia LTR-retrotransposon superfamilies and lineages and their distinct dynamics in the *Populus trichocarpa* (L.) genome. Tree Genet. Genomes 11, 107. 10.1007/s11295-015-0937-z

[B117] Netotea S. Sundell D. Street N. R. Hvidsten T. R. (2014). ComPlEx: conservation and divergence of co-expression networks in *A. thaliana*, *Populus* and *O. sativa*. BMC Genomics 15, 106. 10.1186/1471-2164-15-106 24498971 PMC3925997

[B118] Nielsen R. Paul J. S. Albrechtsen A. Song Y. S. (2011). Genotype and SNP calling from next-generation sequencing data. Nat. Rev. Genet. 12, 443. 10.1038/nrg2986 21587300 PMC3593722

[B119] Noble W. S. (2009). How does multiple testing correction work? Nat. Biotechnol. 27, 1135. 10.1038/nbt1209-1135 20010596 PMC2907892

[B120] Obudulu O. Bygdell J. Sundberg B. Moritz T. Hvidsten T. R. Trygg J. (2016). Quantitative proteomics reveals protein profiles underlying major transitions in aspen wood development. BMC Genomics 17, 119. 10.1186/s12864-016-2458-z 26887814 PMC4758094

[B121] Obudulu O. Mähler N. Skotare T. Bygdell J. Abreu I. N. Ahnlund M. (2018). A multi-omics approach reveals function of secretory carrier-associated membrane proteins in wood formation of *Populus* trees. BMC Genomics 19, 11. 10.1186/s12864-017-4411-1 29298676 PMC5753437

[B122] Ogata Y. Suzuki H. Shibata D. (2009). A database for poplar gene co-expression analysis for systematic understanding of biological processes, including stress responses. J. Wood Sci. 55, 395. 10.1007/s10086-009-1058-9

[B123] O’Malley R. C. Huang S.-s. C. Song L. Lewsey M. G. Bartlett A. Nery J. R. (2016). Cistrome and epicistrome features shape the regulatory DNA landscape. Cell 165, 1280–1292. 10.1016/j.cell.2016.04.038 27203113 PMC4907330

[B124] Paape T. Zhou P. Branca A. Briskine R. Young N. Tiffin P. (2012). Fine-scale population recombination rates, hotspots, and correlates of recombination in the *Medicago truncatula* genome. Genome Biol. Evol. 4, 726–737. 10.1093/gbe/evs046 22554552 PMC3381680

[B125] Patel R. V. Nahal H. K. Breit R. Provart N. J. (2012). BAR expressolog identification: expression profile similarity ranking of homologous genes in plant species. Plant J. 71, 1038–1050. 10.1111/j.1365-313X.2012.05055.x 22607031

[B126] Patti G. J. Yanes O. Siuzdak G. (2012). Innovation: Metabolomics: The apogee of the omics trilogy. Nat. Rev. Mol. Cell Biol. 13, 263. 10.1038/nrm3314 22436749 PMC3682684

[B127] Percival D. Walden A. (2000). Wavelet methods for time series analysis. West Nyack: Cambridge University Press.

[B128] Porth I. Klápště J. Skyba O. Lai B. S. Geraldes A. Muchero W. (2013). *Populus trichocarpa* cell wall chemistry and ultrastructure trait variation, genetic control and genetic correlations. New Phytol. 197, 777–790. 10.1111/nph.12014 23278123

[B129] Quesada T. Li Z. Dervinis C. Li Y. Bocock P. N. Tuskan G. A. (2008). Comparative analysis of the transcriptomes of *Populus trichocarpa* and *Arabidopsis thaliana* suggests extensive evolution of gene expression regulation in angiosperms. New Phytol. 180, 408–420. 10.1111/j.1469-8137.2008.02586.x 18694447

[B130] Ragauskas A. J. Williams C. K. Davison B. H. Britovsek G. Cairney J. Eckert C. A. (2006). The path forward for biofuels and biomaterials. Science 311, 484–489. 10.1126/science.1114736 16439654

[B131] Ritchie M. D. Holzinger E. R. Li R. Pendergrass S. A. Kim D. (2015). Methods of integrating data to uncover genotype–phenotype interactions. Nat. Rev. Genet. 16, 85. 10.1038/nrg3868 25582081

[B132] Sannigrahi P. Ragauskas A. J. Tuskan G. A. (2010). Poplar as a feedstock for biofuels: a review of compositional characteristics. Biofuels Bioprod. Biorefin. 4, 209–226. 10.1002/bbb.206

[B133] Schaefer R. J. Michno J.-M. Myers C. L. (2017). Unraveling gene function in agricultural species using gene co-expression networks. Biochim. Biophys. Acta (BBA)-Gene Regul. Mech. 1860, 53–63. 10.1016/j.bbagrm.2016.07.016 27485388

[B134] Schmid M. Davison T. S. Henz S. R. Pape U. J. Demar M. Vingron M. (2005). A gene expression map of *Arabidopsis thaliana* development. Nat. Genet. 37, 501. 10.1038/ng1543 15806101

[B135] Schönberger B. Chen X. Mager S. Ludewig U. (2016). Site-dependent differences in DNA methylation and their impact on plant establishment and phosphorus nutrition in *Populus trichocarpa*. PloS One 11, e0168623. 10.1371/journal.pone.0168623 27992519 PMC5167412

[B136] Serin E. A. Nijveen H. Hilhorst H. W. Ligterink W. (2016). Learning from co-expression networks: possibilities and challenges. Front. Plant Sci. 7, 444. 10.3389/fpls.2016.00444 27092161 PMC4825623

[B137] Severin A. J. Woody J. L. Bolon Y.-T. Joseph B. Diers B. W. Farmer A. D. (2010). RNA-Seq Atlas of *Glycine max*: a guide to the soybean transcriptome. BMC Plant Biol. 10, 160. 10.1186/1471-2229-10-160 20687943 PMC3017786

[B138] Shannon P. Markiel A. Ozier O. Baliga N. S. Wang J. T. Ramage D. (2003). Cytoscape: a software environment for integrated models of biomolecular interaction networks. Genome Res. 13, 2498–2504. 10.1101/gr.1239303 14597658 PMC403769

[B139] Shi R. Sun Y.-H. Li Q. Heber S. Sederoff R. Chiang V. L. (2009). Towards a systems approach for lignin biosynthesis in *Populus trichocarpa*: transcript abundance and specificity of the monolignol biosynthetic genes. Plant Cell Physiol. 51, 144–163. 10.1093/pcp/pcp175 19996151

[B140] Shim H. Stephens M. (2015). Wavelet-based genetic association analysis of functional phenotypes arising from high-throughput sequencing assays. Ann. Appl. Stat. 9, 655. 10.1214/14-AOAS776 29399242 PMC5795621

[B141] Shuai P. Liang D. Tang S. Zhang Z. Ye C.-Y. Su Y. (2014). Genome-wide identification and functional prediction of novel and drought-responsive lincRNAs in *Populus trichocarpa*. J. Exp. Bot. 65, 4975–4983. 10.1093/jxb/eru256 24948679 PMC4144774

[B142] Shuai P. Liang D. Zhang Z. Yin W. Xia X. (2013). Identification of drought-responsive and novel *Populus trichocarpa* microRNAs by high-throughput sequencing and their targets using degradome analysis. BMC Genomics 14, 233. 10.1186/1471-2164-14-233 23570526 PMC3630063

[B143] Skinner M. E. Uzilov A. V. Stein L. D. Mungall C. J. Holmes I. H. (2009). JBrowse: a next-generation genome browser. Genome Res. 19, 1630–1638. 10.1101/gr.094607.109 19570905 PMC2752129

[B144] Slavov G. T. DiFazio S. P. Martin J. Schackwitz W. Muchero W. Rodgers-Melnick E. (2012). Genome resequencing reveals multiscale geographic structure and extensive linkage disequilibrium in the forest tree *Populus trichocarpa*. New Phytol. 196, 713–725. 10.1111/j.1469-8137.2012.04258.x 22861491

[B145] Slotkin R. K. Martienssen R. (2007). Transposable elements and the epigenetic regulation of the genome. Nat. Rev. Genet. 8, 272. 10.1038/nrg2072 17363976

[B146] Solovieff N. Cotsapas C. Lee P. H. Purcell S. M. Smoller J. W. (2013). Pleiotropy in complex traits: challenges and strategies. Nat. Rev. Genet. 14, 483–495. 10.1038/nrg3461 23752797 PMC4104202

[B147] Spencer C. C. Deloukas P. Hunt S. Mullikin J. Myers S. Silverman B. (2006). The influence of recombination on human genetic diversity. PLoS Genet. 2, e148. 10.1371/journal.pgen.0020148 17044736 PMC1575889

[B148] Suzuki M. M. Bird A. (2008). DNA methylation landscapes: provocative insights from epigenomics. Nat. Rev. Genet. 9, 465. 10.1038/nrg2341 18463664

[B149] Szklarczyk D. Franceschini A. Kuhn M. Simonovic M. Roth A. Minguez P. (2010). The STRING database in 2011: functional interaction networks of proteins, globally integrated and scored. Nucleic Acids Res. 39, D561–D568. 10.1093/nar/gkq973 21045058 PMC3013807

[B150] Szklarczyk D. Morris J. H. Cook H. Kuhn M. Wyder S. Simonovic M. (2017). The STRING database in 2017: Quality-controlled protein–protein association networks, made broadly accessible. Nucleic Acids Res. 45(D1), D362–D368. 10.1093/nar/gkw937 27924014 PMC5210637

[B151] Tang S. Dong Y. Liang D. Zhang Z. Ye C.-Y. Shuai P. (2015). Analysis of the drought stress-responsive transcriptome of black cottonwood (*Populus trichocarpa*) using deep RNA sequencing. Plant Mol. Biol. Rep. 33, 424–438. 10.1007/s11105-014-0759-4

[B152] Taylor G. (2002). Populus Arabidopsis for Forestry. Do We Need a Model Tree?, Ann. Bot. 90 (6), 681–689. 10.1093/aob/mcf255 12451023 PMC4240366

[B153] Thimm O. Bläsing O. Gibon Y. Nagel A. Meyer S. Krüger P. (2004). MAPMAN: a user-driven tool to display genomics data sets onto diagrams of metabolic pathways and other biological processes. Plant J. 37, 914–939. 10.1111/j.1365-313X.2004.02016.x 14996223

[B154] Thurman R. E. Day N. Noble W. S. Stamatoyannopoulos J. A. (2007). Identification of higher-order functional domains in the human ENCODE regions. Genome Res. 17, 917–927. 10.1101/gr.6081407 17568007 PMC1891350

[B155] Tian F. Chang E. Li Y. Sun P. Hu J. Zhang J. (2017). Expression and integrated network analyses revealed functional divergence of NHX-type Na+/H+ exchanger genes in poplar. Sci. Rep. 7, 2607. 10.1038/s41598-017-02894-8 28572621 PMC5453932

[B156] Tschaplinski T. J. Abraham P. E. Jawdy S. S. Gunter L. E. Martin M. Z. Engle N. L. (2019). The nature of the progression of drought stress drives differential metabolomic responses in *Populus deltoides*. Ann. Bot. mcz002. 10.1093/aob/mcz002 PMC6821281 30689716

[B157] Tschaplinski T. J. Plett J. M. Engle N. L. Deveau A. Cushman K. C. Martin M. Z. (2014). *Populus trichocarpa* and *Populus deltoides* exhibit different metabolomic responses to colonization by the symbiotic fungus *Laccaria bicolor*. Mol. Plant-Microbe Interact. 27, 546–556. 10.1094/MPMI-09-13-0286-R 24548064

[B158] Tuskan G. (2007). Bioenergy, genomics, and accelerated domestication: a US example. FAO, Papers and Presentations from The Role of Agricultural Biotechnologies for Production of Bioenergy in Developing Countries, http://www.fao.org/biotech/seminaroct2007.htm.

[B159] Tuskan G. Slavov G. DiFazio S. Muchero W. Pryia R. Schackwitz W. (2011). “Populus resequencing: Towards genome-wide association studies. BMC Proceedings, 5 (Suppl 7), (BioMed Central Ltd), I21. 10.1186/1753-6561-5-S7-I21

[B160] Tuskan G. A. DiFazio S. Faivre-Rampant P. Gaudet M. Harfouche A. Jorge V. (2012). The obscure events contributing to the evolution of an incipient sex chromosome in *Populus*: a retrospective working hypothesis. Tree Genet. Genomes 8, 559–571. 10.1007/s11295-012-0495-6

[B161] Tuskan G. A. Difazio S. Jansson S. Bohlmann J. Grigoriev I. Hellsten U. (2006). The genome of black cottonwood, *Populus trichocarpa* (Torr. & Gray). Science 313, 1596–1604. 10.1126/science.1128691 16973872

[B162] Tuskan G. A. Mewalal R. Gunter L. E. Palla K. J. Carter K. Jacobson D. A. (2018). Defining the genetic components of callus formation: a GWAS approach. PloS One 13, e0202519. 10.1371/journal.pone.0202519 30118526 PMC6097687

[B163] Usai G. Mascagni F. Natali L. Giordani T. Cavallini A. (2017). Comparative genome-wide analysis of repetitive DNA in the genus *Populus* L. Tree Genetics & Genomes 13, 96. 10.1007/s11295-017-1181-5

[B164] Valledor L. Carbó M. Lamelas L. Escandón M. Colina F. J. Cañal M. J. (2018). When the Tree Let Us See the Forest: Systems Biology and Natural Variation Studies in Forest Species. In: Progress in Botany. Springer, Berlin: Heidelberg

[B165] van Eijk K. R. de Jong S. Boks M. P. Langeveld T. Colas F. Veldink J. H. (2012). Genetic analysis of DNA methylation and gene expression levels in whole blood of healthy human subjects. BMC Genomics 13, 636. 10.1186/1471-2164-13-636 23157493 PMC3583143

[B166] Veach A. M. Yip D. Engle N. L. Yang Z. K. Bible A. Morrell-Falvey J. (2018). Modification of plant cell wall chemistry impacts metabolome and microbiome composition in *Populus* PdKOR1 RNAi plants. Plant Soil 429(1–2), 349-361. 10.1007/s11104-018-3692-8

[B167] Verdier J. Torres-Jerez I. Wang M. Andriankaja A. Allen S. N. He J. (2013). Establishment of the *Lotus japonicus* Gene Expression Atlas (LjGEA) and its use to explore legume seed maturation. Plant J. 74, 351–362. 10.1111/tpj.12119 23452239

[B168] Vining K. Pomraning K. R. Wilhelm L. J. Ma C. Pellegrini M. Di Y. (2013). Methylome reorganization during *in vitro* dedifferentiation and regeneration of *Populus trichocarpa*. BMC Plant Biol. 13, 92. 10.1186/1471-2229-13-92 23799904 PMC3728041

[B169] Vining K. J. Pomraning K. R. Wilhelm L. J. Priest H. D. Pellegrini M. Mockler T. C. (2012). Dynamic DNA cytosine methylation in the *Populus trichocarpa* genome: tissue-level variation and relationship to gene expression. BMC Genomics 13, 1. 10.1186/1471-2164-13-27 22251412 PMC3298464

[B170] Visscher P. M. Brown M. A. McCarthy M. I. Yang J. (2012). Five years of GWAS discovery. Am. J. Hum. Genet. 90, 7–24. 10.1016/j.ajhg.2011.11.029 22243964 PMC3257326

[B171] Visscher P. M. Wray N. R. Zhang Q. Sklar P. McCarthy M. I. Brown M. A. (2017). 10 years of GWAS discovery: biology, function, and translation. Am. J. Hum. Genet. 101, 5–22. 10.1016/j.ajhg.2017.06.005 28686856 PMC5501872

[B172] Walley J. W. Sartor R. C. Shen Z. Schmitz R. J. Wu K. J. Urich M. A. (2016). Integration of omic networks in a developmental atlas of maize. Science 353, 814–818. 10.1126/science.aag1125 27540173 PMC5808982

[B173] Wang H. Yan H. Liu H. Liu R. Chen J. Xiang Y. (2018a). GFDP: the gene family database in poplar. Database 2018. 10.1093/database/bay107 PMC6195104 30339216

[B174] Wang J. P. Matthews M. L. Williams C. M. Shi R. Yang C. Tunlaya-Anukit S. (2018b). Improving wood properties for wood utilization through multi-omics integration in lignin biosynthesis. Nat. Commun. 9, 1579. 10.1038/s41467-018-03863-z 29679008 PMC5910405

[B175] Wang L. Xie W. Chen Y. Tang W. Yang J. Ye R. (2010). A dynamic gene expression atlas covering the entire life cycle of rice. Plant J. 61, 752–766. 10.1111/j.1365-313X.2009.04100.x 20003165

[B176] Weckwerth W. (2011). Green systems biology—From single genomes, proteomes and metabolomes to ecosystems research and biotechnology. J. Proteomics 75, 284–305. 10.1016/j.jprot.2011.07.010 21802534

[B177] Weighill D. Jones P. Bleker C. Ranjan P. Shah M. Zhao N. (2019a). Multi-phenotype association decomposition: unraveling complex gene–phenotype relationships. Front. Genet. 10, 417. 10.3389/fgene.2019.00417 31134130 PMC6522845

[B178] Weighill D. Jones P. Shah M. Ranjan P. Muchero W. Schmutz J. (2018). Pleiotropic and epistatic network-based discovery: integrated networks for target gene discovery. Front. Energy Res. 6, 30. 10.3389/fenrg.2018.00030

[B179] Weighill D. Macaya-Sanz D. DiFazio S. P. Joubert W. Shah M. Schmutz J. (2019b). Wavelet-based genomic signal processing for centromere identification and hypothesis generation. Front. Genet. 10, 487 10.3389/fgene.2019.00487 31214244 PMC6554479

[B180] Weighill D. A. Jacobson D. (2017). Network metamodeling: Effect of correlation metric choice on phylogenomic and transcriptomic network topology. Cham: Springer International Publishing, 143–183. 10.1007/10_2016_46 28070594

[B181] Wicker T. Sabot F. Hua-Van A. Bennetzen J. L. Capy P. Chalhoub B. (2007). A unified classification system for eukaryotic transposable elements. Nat. Rev. Genet. 8, 973. 10.1038/nrg2165 17984973

[B182] Wullschleger S. D. Weston D. DiFazio S. P. Tuskan G. A. (2012). Revisiting the sequencing of the first tree genome: *Populus trichocarpa*. Tree Physiol. 33, 357–364. 10.1093/treephys/tps081 23100257

[B183] Xue L.-J. Frost C. J. Tsai C.-J. Harding S. A. (2016). Drought response transcriptomes are altered in poplar with reduced tonoplast sucrose transporter expression. Sci. Rep. 6, 33655. 10.1038/srep33655 27641356 PMC5027551

[B184] Yi F. Jia Z. Xiao Y. Ma W. Wang J. (2018). SPTEdb: A database for transposable elements in salicaceous plants. Database 2018. 10.1093/database/bay024 PMC5846285 29688371

[B185] Yin T. DiFazio S. P. Gunter L. E. Zhang X. Sewell M. M. Woolbright S. A. (2008). Genome structure and emerging evidence of an incipient sex chromosome in *Populus*. Genome Res. 18, 422–430. 10.1101/gr.7076308 18256239 PMC2259106

[B186] Yip A. M. Horvath S. (2007). Gene network interconnectedness and the generalized topological overlap measure. BMC Bioinf. 8, 22. 10.1186/1471-2105-8-22 PMC1797055 17250769

[B187] Zhang B. Horvath S. (2005). A general framework for weighted gene co-expression network analysis. Stat. Appl. Genet. Mol. Biol. 4 (1), 1128. 10.2202/1544-6115.1128 16646834

[B188] Zhang J. Li M. Bryan A. C. Yoo C. G. Rottmann W. Winkeler K. A. (2019). Overexpression of a serine hydroxymethyltransferase increases biomass production and reduces recalcitrance in the bioenergy crop *Populus*. Sustainable Energy Fuels 3, 195–207. 10.1039/C8SE00471D

[B189] Zhang J. Yang Y. Zheng K. Xie M. Feng K. Jawdy S. S. (2018). Genome-wide association studies and expression-based quantitative trait loci analyses reveal roles of HCT 2 in caffeoylquinic acid biosynthesis and its regulation by defense-responsive transcription factors in *Populus*. New Phytol. 220, 502–516. 10.1111/nph.15297 29992670

[B190] Zhang J.-Y. Lee Y.-C. Torres-Jerez I. Wang M. Yin Y. Chou W.-C. (2013). Development of an integrated transcript sequence database and a gene expression atlas for gene discovery and analysis in switchgrass (*Panicum virgatum L*.). Plant J. 74, 160–173. 10.1111/tpj.12104 23289674

[B191] Zhang S. Chen F. Peng S. Ma W. Korpelainen H. Li C. (2010). Comparative physiological, ultrastructural and proteomic analyses reveal sexual differences in the responses of *Populus cathayana* under drought stress. Proteomics 10, 2661–2677. 10.1002/pmic.200900650 20455211

[B192] Zheng K. Wang X. Weighill D. A. Guo H.-B. Xie M. Yang Y. (2016). Characterization of DWARF14 genes in *Populus*. Sci. Rep. 6, 21593. 10.1038/srep21593 26875827 PMC4753501

[B193] Zhou F. Xu Y. (2009). RepPop: a database for repetitive elements in *Populus trichocarpa*. BMC Genomics 10, 14. 10.1186/1471-2164-10-14 19134208 PMC2645430

[B194] Zilberman D. Gehring M. Tran R. K. Ballinger T. Henikoff S. (2007). Genome-wide analysis of *Arabidopsis thaliana* DNA methylation uncovers an interdependence between methylation and transcription. Nat. Genet. 39, 61 10.1038/ng1929. 17128275

