# Supplementary material for: Data Integration in Poplar: ‘Omics Layers and Integration Strategies
Source: Front Genet. 2019 Sep 25;10:874. doi: 10.3389/fgene.2019.00874 (PMC6773870; doi:10.3389/fgene.2019.00874)
Supplement: Supplementary file 1 [file DataSheet_1.pdf]

Supplementary Material:
Data Integration in Poplar: Omics Layers and Integration Strategies

1 GWAS APPROACHES AND MULTIPLE HYPOTHESIS CORRECTION

EMMAX (Kang et al., 2010) is one particular GWAS method that attempts to correct for the effect of individual relatedness within the population. It is a faster version of the EMMA method (Kang et al., 2008). GWAS methods such as EMMAX model the relationship between measured phenotypes and SNPs as a linear model:

$$y_i = \beta_0 + \sum_{k=1}^{M} \beta_k X_{ik} + \epsilon_i \tag{S1}$$

where y_i is a measured phenotype for individual i, β_k is the effect of SNP k on the phenotype, X is a matrix of fixed effects (SNPs) in which X_{ik} is the minor allele count of SNP k in individual i, and ϵ_i represents environmental variation on the phenotype y_i (Kang et al., 2010). The aim is to determine which of the β_k are significantly different from zero, thus identifying which SNPs have a significant effect on the phenotype (Kang et al., 2010). EMMAX accounts for sample structure by calculating a kinship matrix K that contains pairwise genetic similarities of the of the individuals under consideration. A variance component model is used, partitioning the phenotypic variance into variance due to environmental factors σ_e^2, and variance due to the additive effect of genetic factors σ_a^2 (Kang et al., 2010). This variance component model includes the kinship matrix, modeling the variance-covariance structure of the phenotype in terms of the genetic similarity of pairs of individuals defined in the kinship matrix (Kang et al., 2010):

$$\text{Var}(Y) = \sigma_a^2 K + \sigma_e^2 I \tag{S2}$$

where $\text{Var}(Y)$ is the variance-co-variance structure of the phenotype and I is the identity matrix. The β_k are then estimated using Generalized Least Squares and an F-test is used to determine which of these β_k are statistically different from zero (Kang et al., 2010). Each SNP k corresponding with a β_k statistically different from zero thus potentially affects the phenotype. Thus, for a given measured phenotype, EMMAX produces a list of all SNPs and their respective p-values for their association with the phenotype. A p-value threshold can then be applied to determine which of the associations are significant.

Performing a GWAS involves testing multiple hypotheses, each asking "is SNP k associated with the phenotype p?" for each SNP in the dataset. When testing multiple hypotheses, or a so-called family of m hypotheses, the quantity called the Family-wise Error Rate (FWER) becomes inflated (Johnson et al., 2010). The FWER is defined as the probability that at least one null hypothesis was rejected when it should not have been, or, the probability of achieving at least one false positive. When a statistical test is performed and a p-value is generated, the p-value represents the Type-1 error rate (or false-positive rate), which is

the probability that the null hypothesis was incorrectly rejected (Johnson et al., 2010). Let α. represent the originally chosen p-value threshold. Then, for each true null hypothesis, the probability that it was incorrectly rejected is α. Given that a null hypothesis is true, the probability it was not rejected is thus $1 - \alpha$. If we assume that all m null hypotheses are true, the probability that all m null hypotheses were not rejected (i.e. the probability of obtaining no false positives) is $(1 - \alpha)^m$. Therefore, given that all null hypotheses are true, the probability of obtaining at least one false positive (also known as the FWER) is (Johnson et al., 2010):

$$\text{FWER} = 1 - (1 - \alpha)^m \tag{S3}$$

As can be seen from Equation S3, the FWER increases with the number of hypotheses tested. The probability of obtaining false positives thus increases with the number of hypothesis tests performed. Methods for multiple hypothesis correction attempt to control this FWER.

Let $H_1, H_2 ... H_m$ be a family of m hypotheses and let $P_1, P_2 ... P_m$ be their respective p-values. Bonferroni Correction is a simple method which rejects null hypothesis H_i if (Narum, 2006):

$$P_i \leq \frac{\alpha}{m} \tag{S4}$$

This has been proven to control the FWER, ensuring that FWER $\leq \alpha$.

An adaptation to this method known is as Sequential Bonferroni Correction or Holm-Bonferroni Correction (Holm, 1979). This method orders the hypotheses such that $P_1 \leq P_2 \leq ... \leq P_m$. The index k is then determined such that k is the largest index for which the following holds (Holm, 1979):

$$P_k \leq \frac{\alpha}{m + 1 - k} \tag{S5}$$

Hypotheses $H_1, H_2 ... H_k$ are then rejected and hypotheses $H_{k+1}, H_{k+2} ... H_m$ are not rejected.

Another type of multiple hypothesis correction attempts to control the False Discovery Rate (FDR), which is defined as the proportion of rejected null hypotheses that were incorrectly rejected, or, the proportion of Type-1 errors made (Benjamini and Hochberg, 1995). This is performed by ordering p-values in a similar fashion to Holm-Bonferroni Correction. The index k is then determined such that k is the largest index for which the following holds (Benjamini and Hochberg, 1995):

$$P_k \leq \frac{k\alpha}{m} \tag{S6}$$

Hypotheses $H_1, H_2 ... H_k$ are then rejected and hypotheses $H_{k+1}, H_{k+2} ... H_m$ are not rejected. This procedure ensures that the FDR is below α.

point to some reviews

2 NETWORK THEORY

Mathematically, a graph G is an ordered pair defined as $G = (V, E)$ where V is a set of nodes and E is a set of edges (Golumbic, 2004). Each edge $e_{ij} \in E$ is defined as a set of two nodes:

$$e_{ij} = \{i, j\} \tag{S7}$$

where $i \in V$ and $j \in V$. In biological network applications, nodes represent a biological object of interest and edges will represent associations/interactions/similarities between these biological objects.

A graph can be represented numerically as a matrix, namely an Adjacency Matrix (Golumbic, 2004). The Adjacency Matrix A is an $n \times n$ matrix where $n = |V|$, the number of nodes in the network. Each entry a_{ij} in an Adjacency Matrix associated with a graph is defined as:

$$a_{ij} = \begin{cases} 1 & \text{if } \{i, j\} \in E \\ 0 & \text{otherwise.} \end{cases} \tag{S8}$$

The Adjacency Matrix associated with the small example graph in Figure 1A is shown in Figure 1B. Each edge e_{ij} in a graph can be assigned a real number weight w_{ij} which represents the strength of the relationship between the two nodes it connects. A weighted graph can be mathematically represented as a Weighted Adjacency Matrix. This matrix is constructed in a similar manner to the normal Adjacency Matrix. Each entry a_{ij} of the Weighted Adjacency Matrix is defined as:

$$a_{ij} = \begin{cases} w_{ij} & \text{if } \{i, j\} \in E \\ 0 & \text{otherwise.} \end{cases} \tag{S9}$$

where w_{ij} is the weight associated with edge e_{ij} (Golumbic, 2004).

A bipartite graph $G = (V, E)$ is a graph in which the nodes of V can be partitioned into two non-overlapping sets, V_1 and V_2 and each edge $e_{ij} \in E$ is defined as:

$$e_{ij} = \{v_i, v_j\} \tag{S10}$$

where $v_i \in V_1$ and $v_j \in V_2$ (Marcus, 2008). Intuitively, this means that a bipartite graph (or a bipartite network) consists of two classes of nodes in which nodes of one class can only be connected to nodes of the other class. An example of a bipartite network is shown in Figure 1C, and it's matrix representation in Figure 1D.

REFERENCES

Benjamini, Y. and Hochberg, Y. (1995). Controlling the false discovery rate: a practical and powerful approach to multiple testing. *Journal of the Royal Statistical Society. Series B (Methodological)* , 289–300

Golumbic, M. C. (2004). *Algorithmic graph theory and perfect graphs*, vol. 57 (Elsevier)

Holm, S. (1979). A simple sequentially rejective multiple test procedure. *Scandinavian journal of statistics* , 65–70

Johnson, R. C., Nelson, G. W., Troyer, J. L., Lautenberger, J. A., Kessing, B. D., Winkler, C. A., et al. (2010). Accounting for multiple comparisons in a genome-wide association study (GWAS). *BMC genomics* 11, 724

Kang, H. M., Sul, J. H., Service, S. K., Zaitlen, N. A., Kong, S.-y., Freimer, N. B., et al. (2010). Variance component model to account for sample structure in genome-wide association studies. *Nature genetics* 42, 348–354

Kang, H. M., Zaitlen, N. A., Wade, C. M., Kirby, A., Heckerman, D., Daly, M. J., et al. (2008). Efficient control of population structure in model organism association mapping. *Genetics* 178, 1709–1723

Marcus, D. (2008). *Graph theory: A problem oriented approach* (Maa)

Narum, S. R. (2006). Beyond Bonferroni: less conservative analyses for conservation genetics. *Conservation Genetics* 7, 783–787
